# Comparative palatability of orally disintegrating tablets (ODTs) of Praziquantel (L-PZQ and Rac-PZQ) versus current PZQ tablet in African children: A randomized, single-blind, crossover study

**DOI:** 10.1371/journal.pntd.0007370

**Published:** 2021-06-09

**Authors:** Muhidin K. Mahende, Eric Huber, Elly Kourany-Lefoll, Ali Ali, Brooke Hayward, Deon Bezuidenhout, Wilhelmina Bagchus, Abdunoor M. Kabanywanyi

**Affiliations:** 1 Ifakara Health Institute, Dar es Salaam, Tanzania; 2 Swiss Tropical and Public Health Institute, Basel, Switzerland; 3 University of Basel, Basel, Switzerland; 4 Ares Trading S.A., Eysins, Switzerland, an affiliate of Merck KGaA, Darmstadt, Germany; 5 EMD Serono, Inc. Rockland, Massachusetts, United States, an affiliate of Merck KGaA, Darmstadt, Germany; 6 Merck (Pty) Ltd, Modderfontein, South Africa an affiliate of Merck KGaA, Darmstadt, Germany; 7 Merck Institute of Pharmacometrics, Lausanne, Switzerland, an affiliate of Merck KGaA, Darmstadt, Germany; SCI Foundation, AUSTRALIA

## Abstract

**Background:**

Praziquantel (PZQ) is currently the only recommended drug for infection and disease caused by the schistosome species that infects humans; however, the current tablet formulation is not suitable for pre-school age children mainly due to its bitterness and the large tablet size. We assessed the palatability of two new orally disintegrating tablet (ODT) formulations of PZQ.

**Methodology:**

This randomized, single-blind, crossover, swill-and-spit palatability study (NCT02315352) was carried out at a single school in Tanzania in children aged 6–11 years old, with or without schistosomiasis infection as this was not part of the assessment. Children were stratified according to age group (6–8 years or 9–11 years) and gender, then randomized to receive each formulation in a pre-specified sequence. Over 2 days, the children assessed the palatability of Levo-Praziquantel (L-PZQ) ODT 150 mg and Racemate Praziquantel (Rac-PZQ) ODT 150 mg disintegrated in the mouth without water on the first day, and L-PZQ and Rac-PZQ dispersed in water and the currently available PZQ 600 mg formulation (PZQ-Cesol) crushed and dispersed in water on the second day. The palatability of each formulation was rated using a 100 mm visual analogue scale (VAS) incorporating a 5-point hedonic scale, immediately after spitting out the test product (VAS_t = 0_ primary outcome) and after 2–5 minutes (VAS_t = 2–5_).

**Principal findings:**

In total, 48 children took part in the assessment. Overall, there was no reported difference in the VAS_t = 0_ between the two ODT formulations (p = 0.106) without water. Higher VAS_t = 0_ and VAS_t = 2–5_ scores were reported for L-PZQ ODT compared with Rac-PZQ ODT in older children (p = 0.046 and p = 0.026, respectively). The VAS_t = 0_ and VAS_t = 2–5_ were higher for both ODT formulations compared with the standard formulation (p<0.001 for both time points). No serious adverse events were reported.

**Conclusions/Significance:**

The new paediatric-friendly formulations dispersed in water were both found to be more palatable than the existing standard formulation of PZQ. There may be gender and age effects on the assessment of palatability. Further research is needed for assessing efficacy and tolerability of the newly ODTs Praziquantel drug in younger children.

**Trial registration:**

The trial was registered on ClinicalTrials.gov (NCT02315352) and in the Pan African Clinical Trials Registry (PACTR201412000959159).

## Author summary

Schistosomiasis or Bilharzia is among the top debilitating parasitic diseases in endemic developing countries. It presents either in urinary or intestinal form. The diseases’ mode of transmission is waterborne through contact with infested water. The main groups affected in developing countries are women and children due to their frequent contact with water. WHO introduced a mass drug administration program whereby drugs are distributed in endemic communities to cut off the transmission of NTDs including schistosomiasis.Praziquantel is the sole drug for the treatment of all forms of Schistosomiasis currently and it has been proven to be highly efficacious. The WHO’s Preventative chemotherapy program uses the same drug as a prophylactic tool to control the disease.This drug presents a challenge in administering it to younger school children due to its size and the unpleasant taste of the available 600mg tablet. This makes it difficult to administer the correct dosage of the drug to school children and it excludes preschoolers from administration program.This study was done as a swillandspit exercise (the drug was not ingested) to assess the new orally disintegrating isomers of Praziquantel, L-PZQ and Rac-PZQ which have been prepared as 150mg tablets and sweetened through the addition of a sweetener (Sucralose), in comparision to the existing Praziquantel formulation. Findings from 48 African children showed that both new formulations are more palatable to younger children as compared to the existing Praziquantel formulation.These results provided confidence for further evaluation of the clinical efficacy and tolerability of the paediatric friendly formulations of Praziquantel tablets for schistosomiasis treatment.

## Introduction

Schistosomiasis is a Neglected Tropical Disease and is one of the major parasitic diseases of public health importance that causes morbidity and mortality annually in endemic countries[1]. Globally, about 200 million people are infected each year and 40% of these cases are reported in sub-Saharan Africa alone[2,3]. Schistosomiasis is second to malaria as the parasitic cause of severe morbidities in endemic areas Sub-Saharan Africa[4–6]. The three main species of the Genus *Schistosoma* that infect humans are *Schistosoma haematobium*, which causes urogenital schistosomiasis, as well as *Schistosoma japonicum* and *Schistosoma mansoni*, which cause intestinal schistosomiasis[7].

The disease burden worldwide does not exclude younger children, especially in endemic areas[8]. Over 123 million children are estimated to suffer from schistosomiasis globally causing significant health problems, with negative impacts on children’s growth and development[9,10]. This predisposes these younger children to several pathological problems like nutritional deficits,[7] anaemia, as well as impairment in their neuro-cognitive development[11–13]. Further serious consequences of schistosomiasis have far-reaching complications in multiple human organ systems, including irreversible pulmonary hypertension, renal, genitourinary, central nervous system and neoplastic conditions[14].

The disease is most prevalent in tropical and sub-tropical areas, poor communities that are subject to unreliable water sources and inadequate sanitation[8]. Studies that have been done in several parts of Tanzania showed varying high burden (>80%) of urinary and intestinal schistosomiasis in children[15,16]. These studies revealed that school children and preschoolers bear a significant burden and are at greater risk of being infected due to poor personal and environmental hygiene[17,18]. Another study by Mwinzi et al (2015) in Kenya showed that the prevalence and intensity of Schistosoma infections are comparatively higher in younger children than in other population groups in communities[19].

Since the 54th World Health Assembly in 2001, WHO has been using Praziquantel (PZQ) in anti-helminthic deworming campaigns for Mass Drug Administration (MDA) against schistosomiasis globally,[20] observing high success rate in both children and adults as reported by community-based surveys[21]. Since 2000, several programs have been set up globally in NTD endemic countries including Tanzania to reduce schistosomiasis and other related neglected tropical diseases in communities with high prevalence[22–24]. In 2009 the country operationalized the WHO campaign program of annual and biannual mass drug administration for prevention and control of NTDs including Schistosomiasis, in adults and school-age children[25,26].

However, pre-school children were still not included in the deworming program in all endemic countries delivering MDA and there are no specified recommendations on the inclusion of very young children in mass treatment campaigns[27,28]. This was due to the lack of enough data on the safety of the drug as to date there is no appropriate paediatric formulation of PZQ currently available for both schoolers and the preschoolers children[9,29]. Several studies have demonstrated fewer side effects, efficacy (parasite clearance), and good tolerability of preschoolers when given crushed PZQ drugs for treatment. This provides evidence for the newly formulated PZQ to allow the inclusion of this group in MDA campaigns and in treatment as well[30–32].

Praziquantel is the only currently available anti-schistosome drug that is recommended for the species of schistosome that infects humans[1]. Although it is widely used for treatment, it is also highly effective when used as prophylaxis[33]. A phase II efficacy study in Ivory Coast has suggested that both ODT formulations are well tolerated at all doses tested in children aged 6 years and above; one formulation, Levo-praziquantel (L-PZQ) ODT has been confirmed for further development[34].

Despite the wide use of PZQ, treatment compliance in children is hindered by the bitter taste of the formulation and the size of the tablet[35]. The development of a paediatric-friendly PZQ formulation that is not bitter to taste will increase the uptake of medication among young-age populations, will help to improve treatment in routine care settings and enable the inclusion of preschoolers in mass drug administration campaigns[5].

To address this public health question, the Paediatric Praziquantel Consortium was formed in July 2012 to develop an ODT formulation of PZQ for the treatment of pre-school age children with schistosomiasis and obtain regulatory approval of this formulation to allow future access. Ifakara Health Institute (IHI, Tanzania), in collaboration with the Consortium carried out this trial, which evaluated the palatability of two new ODT formulations of PZQ in comparison with the currently available PZQ tablets (PZQ-Cesol)[29,36].

## Methods

### Ethics statement

Ethical clearance was obtained from the Ifakara Health Institute review board (IHI/IRB/NO:40–2014), the National Medical Research Council Committee (MRCC)-NIMR (Ref No. NIMR/HQ/R8.a/Vol. IX/1872), and the ethical committee of North-Western and Central Switzerland (EKNZ; approval number EKNZ2014-400). The trial protocol and the medicinal products were further approved by the Tanzanian food and drug authority (TFDA; approval number TZ15CT006). The data safety monitoring committee was established by the sponsor.

Children and their parents were notified about the trial through an open community-based recruitment process, during which investigators conducted community-based meetings, and the parents of school children and local community leaders were invited to participate during meetings on the school premises. Detailed information about the study procedures and other trial-related requirements (e.g., consent forms) were provided to those who were willing to participate, and written consent forms were also shared with Umwe primary school teachers for them to verify the information with parents and children. All parents gave their written informed consent for the child to take part and the child gave written assent that they were willing to take part and to comply with the trial procedure. Standard Praziquantel (Cesol-PZQ) drugs were freely given to all community members at the end of the trial.

### Study design and participants

This was a randomized, single-blind, five-period crossover clinical trial done as a swill-and-spit palatability assessment of the 2 new paediatric-friendly ODT formulations of PZQ vs. the currently available PZQ tablet carried out in a single school on two consecutive days over one weekend. Each participant sampled each formulation/preparation.

On Day 1, the participants assessed the palatability of the following arms in a randomized sequence:

- L-PZQ ODT (150 mg) put and disintegrated in the mouth
- Rac- PZQ ODT (150 mg) put and disintegrated in the mouth

On Day 2, the same participants assessed the palatability of the following arms in a randomized sequence:

C L-PZQ ODT (150 mg) dispersed in water administered in the mouth cavity
D Rac- ODT (150 mg) dispersed in water administered in the mouth cavity
E 150 mg current PZQ tablet (1/4 of a 600 mg tablet) crushed, dispersed in water and administered in the mouth cavity.

Randomization resulted in 12 sequences: ABCDE, ABCED, ABDCE, ABDEC, ABECD, ABEDC, BACDE, BACED, BADCE, BADEC, BAECD, and BAEDC. Eligible participants were enrolled in one of the four stratification groups (males ages 6–8, males ages 9–11, females ages 6–8 and females ages 9–11 years old) until there were 12 in each group and then randomized to one of the 12 sequences. In this way, equal numbers of each sex and each age category received each formulation in each sequence. Recruitment was done from classes I–V at Umwe primary school in Rufiji district located in south-east Tanzania, where schistosomiasis is endemic.

Children of either gender, with or without history of schistosomiasis as this was not assessed, were eligible if they were aged 6–11 years, inclusive (age was ascertained from the parents verbally and verified from a legal birth certificate or school records), they were able to comply with the protocol, and they could communicate well with the investigator in Swahili language. Children were excluded if they were not able to attend the follow-up visits, had any pre-existing condition or dietary habit that was known to interfere with their sense of smell, taste or ingestion of medication, were febrile or had a history of body temperature ≥38°C, had a respiratory and/or heart rate higher than normal, had the presence of oral thrush in the 2 weeks preceding the trial, or were in any other clinical investigation for any other pharmaceutical product in the preceding 4 weeks.

### Screening

**Fig 1:**
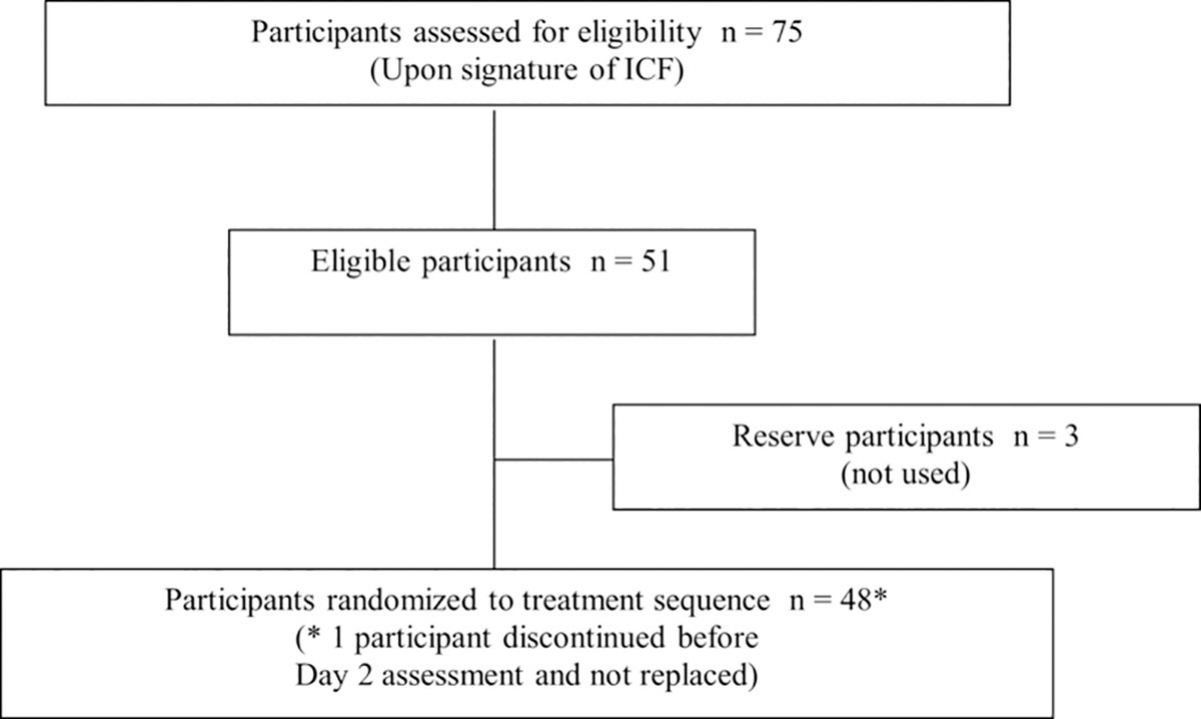
Flow diagram of participant distribution showing the number of participants assessed for eligibility enrolled and randomized.

Participants were invited to come to the school with their parents, where all screening procedures, training and further recruitment were carried out. They were screened for their ability to perform the taste assessment and instructed on how to adhere to the taste trial procedures and regulations. The screening was completed in four rounds over two consecutive weeks on consecutive days over the weekend. Children were routinely assessed for their ability to hold 2 mL of juice in their mouth for 10 seconds and then spit it out as well as their ability to keep candy in their mouth for 20 seconds without swallowing it. They were asked to properly assess and differentiate the flavors of different drinks (e.g., mango or orange juice) and to give feedback. Those 75 who passed these preliminary tests were instructed how to use a smiley and dismal presentation of the pictorial hedonic scale to describe their feeling after every gustatory test. Children were screened until 48 children could be included in 4 equal-sized groups by age (6–8 years or 9–11 years) and gender, n = 12 per group (Fig 1). These 48 children were provided with notification letters to take home on Fridays to alert parents and guardian on attending the next day for assessment and a phone call was further made for more emphasis on attendance. These children were instructed and observed to hold an investigational medicinal product (IMP) either a tablet or dispersed solution for 10 seconds in the mouth and spit out immediately as instructed by a study team member. All the recruited children in this study were able to hold the IMP for 10 seconds without swallowing or spit out before 10 seconds as instructed. Neither a child was reported to spit before the recommended time of 10 seconds nor swallow the IMP.

### Test procedures and medications

Test assessments were carried out within the school premises during weekends in April and May 2015. The investigation medicinal product (IMP); L-PZQ ODT, Rac-PZQ ODT or the currently available PZQ tablet-Cesol formulation) was prepared by a pharmacist in a separate room, to which the rest of the investigation team had limited access. The study pharmacist who was issuing the investigational product was the only person who was aware of the formulation given to a particular participant. On Day 1, participants assessed the palatability of the following arms in a randomized sequence: A) L-PZQ ODT (150 mg) disintegrated in the mouth and B) Rac-PZQ ODT (150 mg) disintegrated in the mouth. On Day 2, participants assessed the palatability of the following arms in a randomized sequence: C) L-PZQ ODT (150 mg) dispersed in water; D) Rac-PZQ ODT (150 mg) dispersed in water; E) current PZQ tablet (150 mg, 1/4 of a 600 mg tablet) crushed and dispersed in water. Randomization of these 5 periods resulted in 12 sequences: ABCDE, ABCED, ABDCE, ABDEC, ABECD, ABEDC, BACDE, BACED, BADCE, BADEC, BAECD, and BAEDC. The test sequence was determined from a dedicated randomization list stratified by age group and gender. Participants tasted each preparation only once in each of the five periods. There were 2 hours between each assessment.

**Fig 2:**
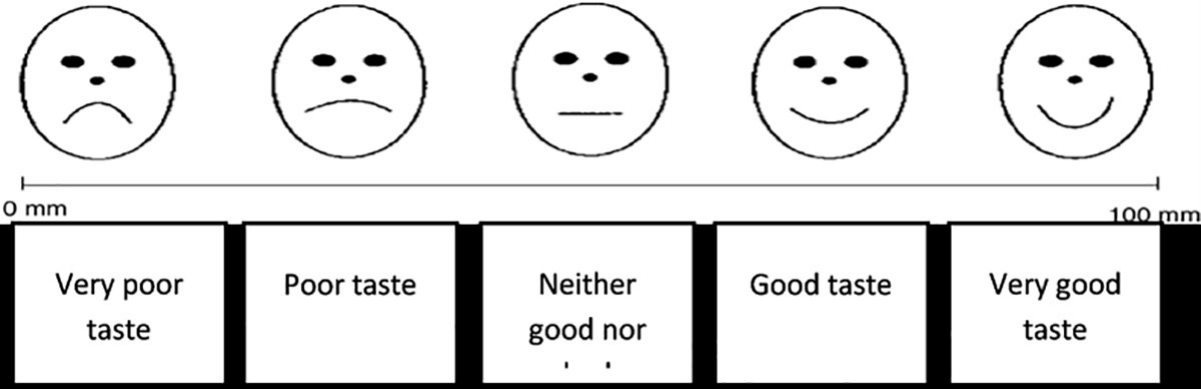
100 mm Hedonic VAS scale.

Following each swill-and-spit assessment, the participants were directed to an assessment room separate from the test room. In this room clinicians administered separate questionnaires incorporating the pictorial hedonic scale for the children to report their taste opinions from each assessment period. The tool employed in this study has been widely used in palatability studies in children and is recommended by the European Medicines Agency to be used for research in children. European Medicines Agency recommended the use of hedonic scales and verbal judgment as standard evaluation techniques for younger children below 4 years as they tend to be shy, reluctant, unable to concentrate and also unable to give their taste preferences[37–39] The hedonic scale used bears 5 facial emoji pictorials placed along the line with a scale of 100mm[40,41] The scale was ranging from the very poor taste at 0 mm mark to very good taste at 100 mm mark (Fig 2). Children were asked to place a mark along the line of the 100 mm visual analogue scale (VAS) that incorporated a 5-point hedonic scale for overall palatability. Besides, any discomfort or other occurrences concerning the taste of the study medication (e.g., that led to the medicine being spat out) were also recorded. An open-ended questionnaire to describe the feeling in the mouth and the taste was completed for each child during the washout period between tests.

To standardize the gustatory environment and ensuring children were not hungry between assessments, they were given a breakfast 2 hours before the first assessment. Immediately after each assessment, a cracker with a neutral smell and taste was given to all children, to remove any residual taste in the mouth. The crackers that used were specially made up of plain wheat flour with neither any sweetener nor flavor added which would have elicited any notable smell or taste. Children were kept under the supervision of an investigator to ensure they did not eat anything between test periods.

### Primary outcome

The first primary outcome was the difference in VAS score taken at 0 minutes immediately after spitting out (VAS_t = 0_) for L-PZQ ODT disintegrated in the mouth without water versus Rac-PZQ ODT disintegrated in the mouth without water on Day 1.

### Secondary outcome

The secondary outcome was the difference in VAS score taken at 0 minutes immediately after spitting out (VAS_t = 0_) for L-PZQ ODT (150 mg) dispersed in water or Rac-PZQ ODT (150 mg) dispersed in water versus the current PZQ-Cesol tablet (150 mg, 1/4 of a 600 mg tablet) crushed and dispersed in water on Day 2. Other secondary outcomes were the VAS scores taken at 2 to 5 minutes (VASt_2–5_) after spitting out the IMP on Day 1 and Day 2.

### Other outcomes

Other outcomes included the recording of any discomfort or another observation was done throughout the study duration. As this was only a swill-and-spit study, no other safety profile or tolerability assessments like blood tests were evaluated. Participants were instructed not to swallow the IMP, and even in a situation of inadvertent swallowing or high buccal absorption of the 150 mg tablet, which is one-quarter of a full tablet in solution, the low dose presented a negligible risk of drug effect.

### Statistical analysis

The crossover study design was chosen to allow for a smaller sample size as participants were able to assess all 5 formulations/preparations while acting as their own control. To ensure children of both genders and different age groups (6–8 years or 9–11 years) were represented and to balance the treatment sequences on Day 2 (which required 6 sequences to randomize the 3 formulations given), the sample size was required to be a multiple of 24. We determined *a priori* that a total sample size of 48 participants was considered to have adequate power(>80%) to fulfil the primary and secondary objectives of the study. On Day 1, 48 subjects would provide at least 89.2% power to detect a mean difference in palatability of 10 points in VAS score between Rac-PZQ ODT and L-PZQ ODT without water, assuming a within-subject standard deviation of 15 points (square root of within subject Mean Squared Error [MSE] from the crossover analysis of variance) and a two-sided Type 1 error of 5.0%. On Day 2, 48 subjects would provide at least 89.5% power to detect a mean difference in palatability of 11 points in VAS score between one of the new formulations Rac-PZQ or L-PZQ and the current PZQ formulation crushed in water, assuming a within-subject standard deviation of 15 points (square root of within subject MSE) and a two-sided Type 1 error of 2.5% (chosen as alpha = 5% divided by 2, to adjust for the multiplicity). Calculations were based on a two-sample t-test for the difference in means using crossover data (East Software, version 6.2, Cytel, Cambridge, MA USA). Participants were enrolled into 4 equal-sized groups of 12 according to age category (6–8 years or 9–11 years) and gender and then randomized to one of 12 sequences such that each formulation/preparation had equal numbers of each gender and each age category for each sequence.

Study data were collected using paper-based case report forms (CRF) and then double-entered in OpenClinica open-source software.

Data were to be analyzed using absolute and relative frequencies for qualitative variables or means (standard deviation [SD]) or median (interquartile range [IQR], minima and maxima [min, max]) for quantitative variables. Descriptive analyses of outcome variables were stratified by formulation/preparation group and time of assessment. The VAS was summarized using descriptive statistics according to the formulation/preparation group.

To test the palatability, it was hypothesized that Rac-PZQ ODT (without water) and L-PZQ ODT (without water) taste different (H_A1_), as opposed to the null hypothesis (H_01_) that the tastes of the two formulations are indistinguishable. This was assessed using a linear mixed model with a factor variable for the two formulations and with random intercepts at the level of the children. The second hypothesis (H_A2_) was that at least one of the two new formulations (with water) differs in taste from the current PZQ-Cesol formulation. Here, each of the new formulations was tested against the current formulation at alpha = 0.025. The test sequence, gender, and age of the participants were considered when testing these hypotheses.

Linear mixed modelling was also used to determine the association among VAS scores and gender and age, and to identify potential interactions of these factors with the taste of the formulations. Backward model selection was conducted guided by the Akaike information criterion (AIC). The model with the lowest AIC value that included at least the independent factors (sequence, formulation, gender, and age) was selected as the final model. P-values were considered significant at the two-sided alpha = 0.05 level with no adjustment for multiple comparisons across models. The analysis was completed using R software (version 3.1.2. Vienna, Austria)

## Results

### Participants

During the screening, 75 children who had undergone repeated training rounds on how to adhere to study procedures were identified. Of these, 48 children were deemed eligible for participation distributed in equal-sized groups according to age group and gender and randomized such that equal numbers received each test sequence in each group. An additional 3 eligible participants were identified as reserve participants in case one of the first 48 participants withdrew but were not used. The weight of the participants ranged from 14.7 to 33.1 kg, corresponding to PZQ doses of 4.5 to 10.2 mg/kg if the 150 mg dose were to be unintentionally swallowed during the process. Mean (SD) body temperature, respiratory rates and heart rates were within normal ranges before and after each assessment (Table 1).

**Table 1:** Vital signs by visit.

Day of observation	N participants	Heart rate, beats per minute Mean (SD)	Temperature, C° Mean (SD)	Respiratory rate, breaths per minute Mean (SD)
At screening	48	87.0 (6.23)	36.5 (0.34)	22.5 (4.03)
Day 1 before assessment	48	85.1 (6.19)	36.3 (0.24)	19.5 (1.92)
Day 1 after assessment	48	85.3 (5.51)	36.5 (0.26)	19.3 (1.33)
Day 2 before assessment	47	85.6 (5.62)	36.3 (0.24)	19.5 (1.63)
Day 2 after assessment	47	85.6 (4.93)	36.5 (0.28)	19.3 (1.58)

SD, standard deviation.

### Primary outcome

The overall mean (SD) VAS_t = 0_ scores in millimeters (both age groups and genders combined) for L-PZQ ODT and Rac-PZQ ODT without water were 49.0 (33.7) and 39.3 (28.4), respectively. The mean difference (SD) in VAS_t = 0_ scores between the two formulations was not significant (9.6 [40.3]; p = 0.106) (Table 2).

**Table 2:** VAS scores (mm) for L-PZQ ODT and Rac-PZQ ODT without water (Day 1) at time points 0 and 2–5 minutes and stratified by age category.

Formulation	At time 0 minutes		At time 2–5 minutes	
	Mean (SD)	*p*-value	Mean (SD)	*p*-value
All participants, n = 48				
L-PZQ ODT	49.0 (33.65)		50.7 (27.23)	
Rac-PZQ ODT	39.3 (28.43)		45.1 (29.62)	
Difference (L-Rac)	9.6 (40.29)	0.106^a^	5.6 (36.15)	0.284^b^
Among 6–8 years old, n = 24				
L-PZQ ODT	49.6 (38.28)		51.5 (25.96)	
Rac-PZQ ODT	45.8 (33.14)		56.2 (28.81)	
Difference (L-Rac)	3.8 (44.11)	0.680^c^	-4.7 (37.42)	0.555^c^
Among 9–11 years old, n = 24				
L-PZQ ODT	48.3 (29.11)		50.0 (28.97)	
Rac-PZQ ODT	32.8 (21.60)		34.0 (26.60)	
Difference (L-Rac)	15.5 (36.07)	0.046^c^	15.9 (32.39)	0.026^c^

SD, standard deviation

^a^From linear mixed model adjusted for a period, gender and age group. No interactions were retained in the model.

^b^From linear mixed model adjusted for a period, gender, age group and interaction terms for age with gender and age with the formulation.

^c^From linear mixed model adjusted for period and gender. No interactions were retained in the model.

### Secondary outcomes

#### L-PZQ ODT and Rac-PZQ ODT without water

**Fig 3:**
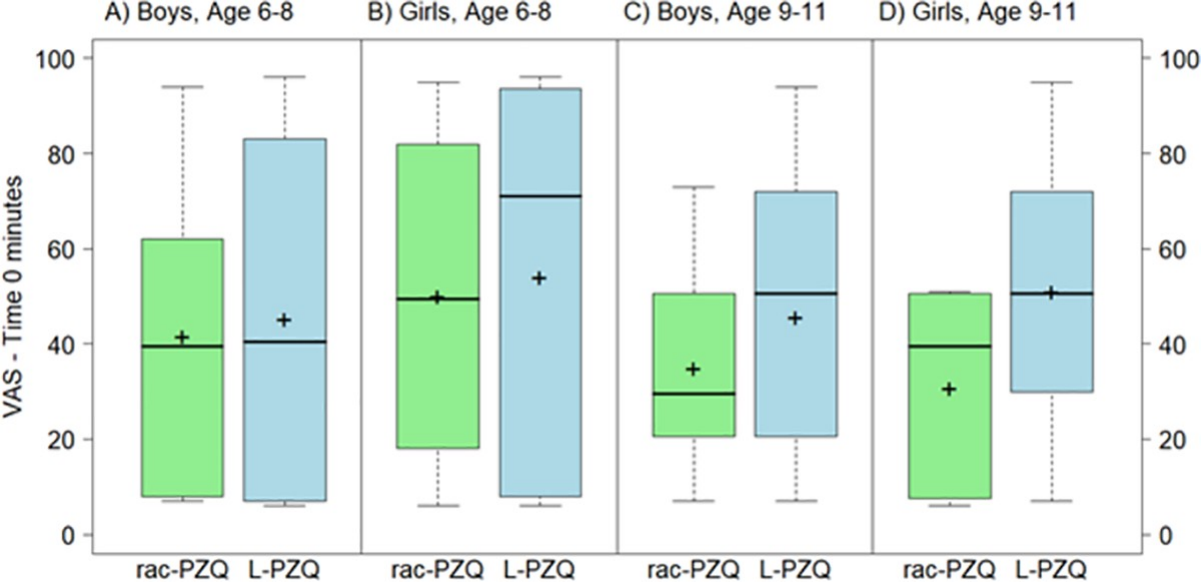
Boxplot of VAS scores (mm) for L-PZQ ODT and Rac-PZQ ODT without water at time 0 minutes, by gender and age. Dark lines indicate median values; ’+’ indicate mean values; the boxes represent the middle 50% of data with the bottom and top of the box represent the first and third quartiles (interquartile range, IQR); the whisker lines above and below the boxes represented the largest and smallest values that are not considered to be outliers; outliers are values that are 1.5 times the IQR below the first quartile or above the third quartile and are denoted with an open circle.

**Fig 4:**
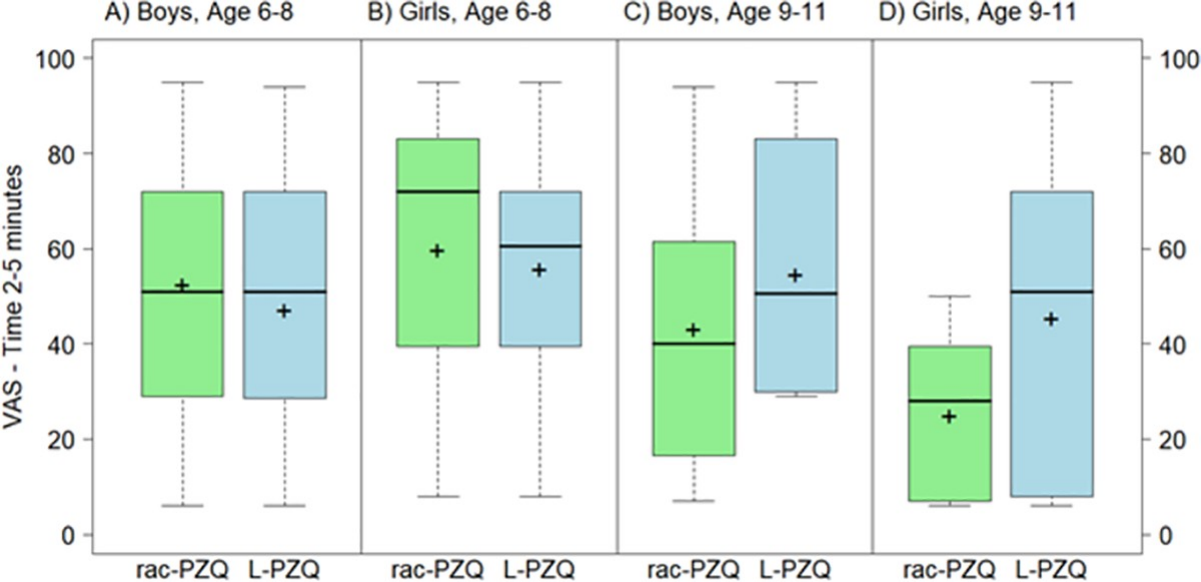
Boxplot of VAS scores (mm) for L-PZQ ODT and Rac-PZQ ODT without water at time 2–5 minutes, by gender and age. Dark lines indicate median values; ’+’ indicate mean values; the boxes represent the middle 50% of data with the bottom and top of the box represent the first and third quartiles (interquartile range, IQR); the whisker lines above and below the boxes represented the largest and smallest values that are not considered to be outliers; outliers are values that are 1.5 times the IQR below the first quartile or above the third quartile and are denoted with an open circle.

The overall mean (SD) VAS_t = 2–5_ scores for L-PZQ ODT and Rac-PZQ ODT without water were 50.7 (27.2) and 45.1 (29.6), respectively. The overall mean (SD) difference in VAS_t = 2–5_ scores between the two formulations was not significant (5.6 [36.1]; p = 0.284). However, there was a significant interaction between the formulation and age group at this time point (interaction p = 0.0499). The mean VAS scores for L-PZQ ODT without water did not differ from scores for Rac-PZQ ODT without water in younger children (6–8 years old). Among older children (9–11 years old), VAS scores for L-PZQ ODT without water compared with Rac-PZQ ODT without water were higher by more than 15 mm (p = 0.046 and p = 0.026 at time 0 and 2–5 minutes, respectively) (Table 2). The distributions of VAS_t = 0_ and VAS_t = 2–5_ scores by gender and age group for the ODT formulations without water are shown in Figs 3 and 4.

#### L-PZQ ODT, Rac-PZQ ODT and current PZQ-Cesol with water

**Fig 5:**
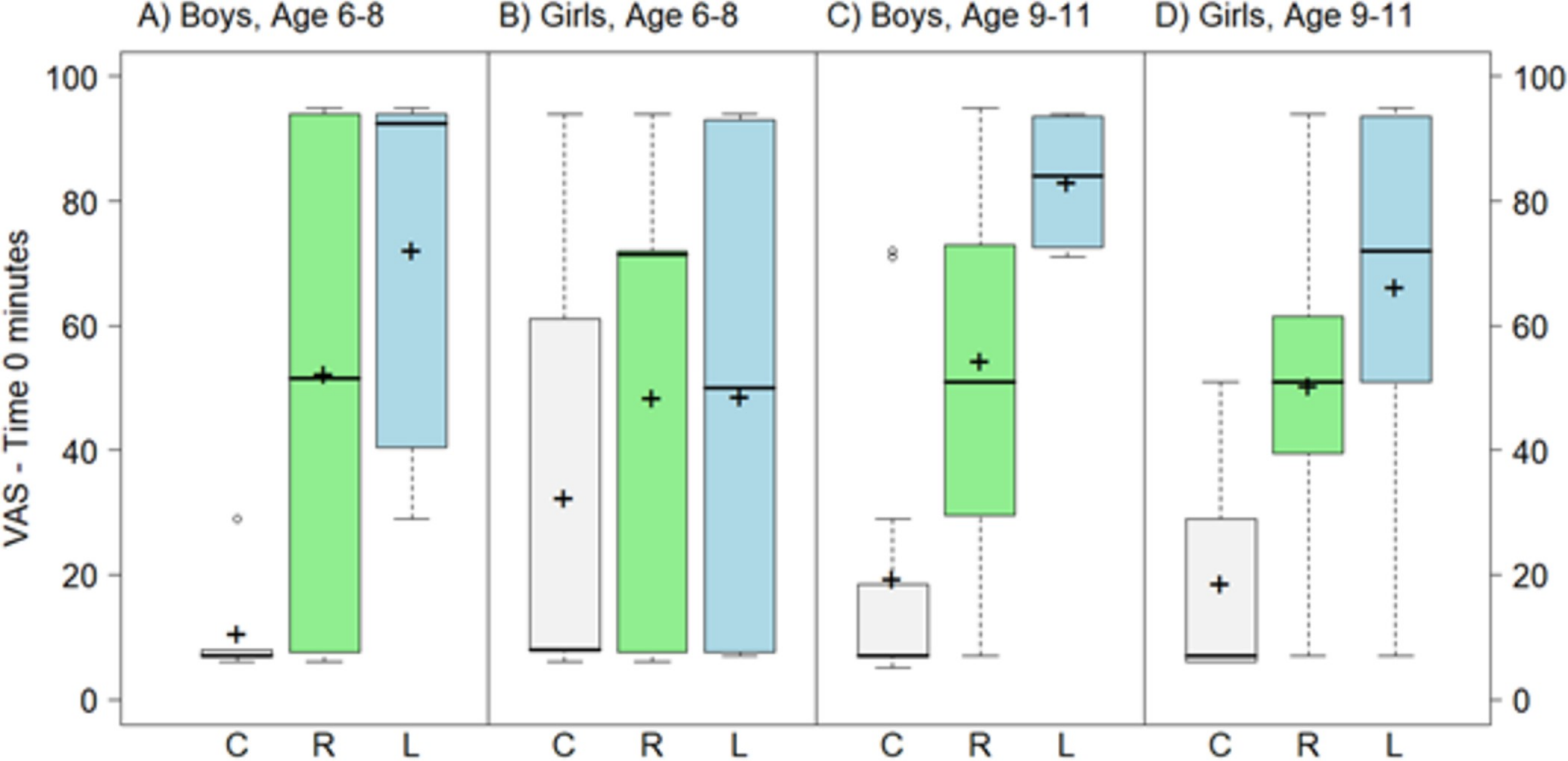
Boxplot of VAS scores (mm) for L-PZQ ODT (L) and Rac-PZQ ODT (R) dispersed with water and current PZQ-Cesol (C) crushed in water at time 0 minutes, by gender and age. Dark lines indicate median values; ’+’ indicate mean values; the boxes represent the middle 50% of data with the bottom and top of the box represent the first and third quartiles (interquartile range, IQR); the whisker lines above and below the boxes represented the largest and smallest values that are not considered to be outliers; outliers are values that are 1.5 times the IQR below the first quartile or above the third quartile and are denoted with an open circle.

**Fig 6:**
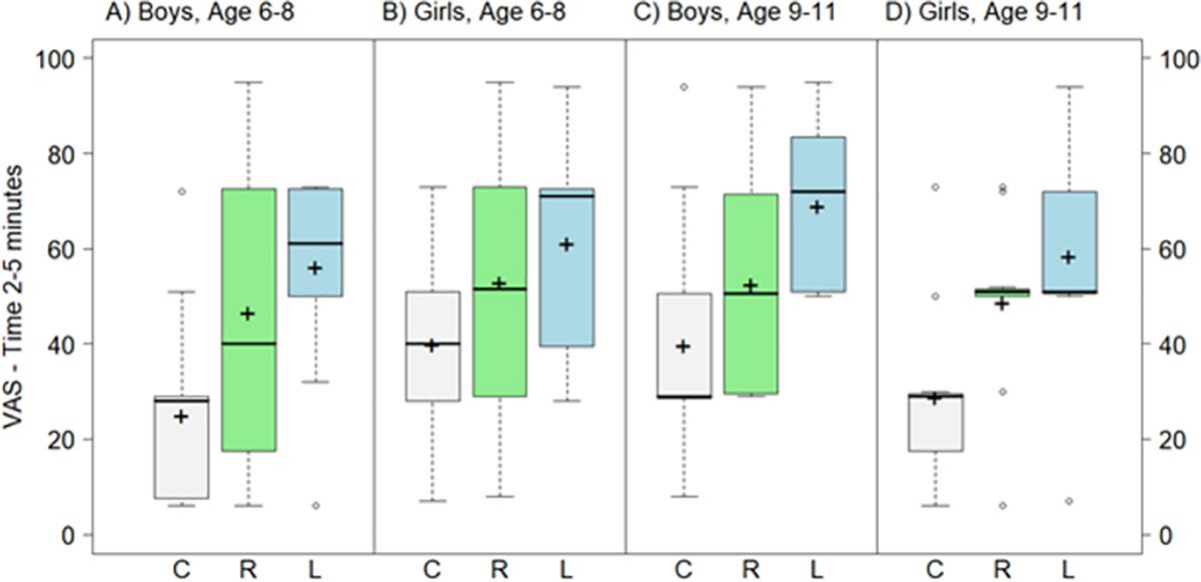
Boxplot of VAS scores (mm) for L-PZQ ODT (L) and Rac-PZQ ODT (R) dispersed with water and current PZQ-Cesol (C) crushed in water at time 2–5 minutes, by gender and age. Dark lines indicate median values; ’+’ indicate mean values; the boxes represent the middle 50% of data with the bottom and top of the box represent the first and third quartiles (interquartile range, IQR); the whisker lines above and below the boxes represented the largest and smallest values that are not considered to be outliers; outliers are values that are 1.5 times the IQR below the first quartile or above the third quartile and are denoted with an open circle.

The overall mean (SD) VAS_t = 0_ score for current PZQ-Cesol crushed in water was 20.3 (24.9) mm, while for L-PZQ ODT dispersed in water it was 67.5 (31.1) mm and for Rac-PZQ ODT dispersed in water it was 51.4 (32.4) mm. Similarly, at 2–5 minutes, the overall mean (SD) VAS_t = 2–5_ scores were 33.5 (22.2) for current PZQ-Cesol, 61.1 (21.2) for L-PZQ ODT and 50.2 (25.9) for Rac-PZQ ODT in water. The differences in VAS scores for L-PZQ ODT and Rac-PZQ ODT dispersed in water compared with PZQ-Cesol were higher at both time points (p<0.001; Table 3). Besides, there was an interaction between the L-PZQ ODT formulation and gender (interaction p = 0.004) with higher VAS_t = 0_ scores for L-PZQ ODT in boys. When stratifying by gender, VAS scores for L-PZQ ODT and Rac-PZQ ODT dispersed in water were also higher than for PZQ-Cesol at each time point (p<0.01; Table 3). The distributions of VAS_t = 0_ and VAS_t = 2–5_ scores by gender and age group are shown in Figs 5 and 6.

**Table 3:** VAS scores (mm) for L-PZQ ODT dispersed in water and Rac-PZQ ODT dispersed in water relative to current PZQ-Cesol crushed in water, at time 0 and 2–5 minutes and stratified by gender.

Formulation	At time 0 minutes		At time 2–5 minutes	
	Mean (SD)	*p*-value	Mean (SD)	*p*-value
All participants, n = 47				
L-PZQ ODT	67.5 (31.14)		61.1 (21.19)	
Rac-PZQ ODT	51.4 (32.35)		50.2 (25.92)	
PZQ-Cesol	20.3 (24.91)		33.5 (22.23)	
Difference (L vs. Cesol)	47.2 (38.27)	<0.001^a^	27.7 (23.58)	<0.001^b^
Difference (Rac vs. Cesol)	31.1 (38.13)	<0.001^a^	16.7 (30.15)	<0.001^b^
Difference (L vs. Rac)	16.1 (37.46)	0.002^a^	11.0 (27.50)	0.008^b^
Among Boys, n = 24				
L-PZQ ODT	77.6 (22.02)		62.5 (20.25)	
Rac-PZQ ODT	53.3 (34.23)		49.5 (27.60)	
PZQ-Cesol	15.0 (18.92)		32.4 (23.64)	
Difference (L vs. Cesol)	62.6 (27.23)	<0.001^c^	30.1 (26.40)	<0.001^c^
Difference (Rac vs. Cesol)	38.3 (41.70)	<0.001^c^	17.1 (31.43)	0.005^c^
Difference (L vs. Rac)	24.3 (31.39)	0.001^c^	13.0 (26.20)	0.010^c^
Among Girls, n = 23				
L-PZQ ODT	57.0 (35.99)		59.7 (22.48)	
Rac-PZQ ODT	49.4 (30.89)		50.9 (24.63)	
PZQ-Cesol	25.9 (29.34)		34.6 (21.12)	
Difference (L vs. Cesol)	31.2 (41.97)	<0.001^c^	25.2 (20.52)	<0.001^c^
Difference (Rac vs. Cesol)	23.6 (33.26)	0.007^c^	16.3 (29.45)	0.005^c^
Difference (L vs. Rac)	7.6 (41.89)	0.431^c^	8.8 (29.23)	0.181^c^

SD, standard deviation

^a^From linear mixed model adjusted for a period, gender, age group and an interaction term for gender with the formulation.

^b^From linear mixed model adjusted for a period, gender and age group. No interactions were retained in the model.

^c^From linear mixed model adjusted for period and age group. No interactions were retained in the model.

#### L-PZQ ODT and Rac-PZQ ODT with water

Both the mean VAS_t = 0_ and VAS_t = 2–5_ for L-PZQ ODT dispersed in water were significantly higher than for Rac-PZQ ODT dispersed in water (16.1 [37.5] and 11.0 [27.5], respectively, p<0.01 for each time point; Table 3). When stratifying by gender, only VAS scores for boys remained significantly higher for L-PZQ ODT compared with Rac-PZQ ODT when dispersed in water.

### Adverse events

There were no severe adverse events observed throughout the trial period. Two (4.2%) participants reported occurrences of discomfort each during the study. One child reported abdominal discomfort on Day 2; this child had the Day 2 sequence of Rac-PZQ ODT/current PZQ-Cesol/L-PZQ ODT. Another child reported a headache on Day 2; this child had the Day 2 sequence of Rac-PZQ ODT/L-PZQ ODT/PZQ-Cesol. One child (2.1%) presented with malaria, which was constitutionally considered an adverse event before tasting on Day 2. This child was removed from the study and treated with appropriate antimalarial medication; she was followed up and was not replaced in the study with another child.

## Discussion

When the two ODT formulations were tasted without water, mean VAS scores were acceptable in the “Neither good nor bad” range of the hedonic VAS scale and the VAS score for L-PZQ ODT did not differ from that for Rac-PZQ ODT. Overall, the between-participant variability was considerable, especially for age and gender. Although limited to relatively small sample sizes of 24 participants each, analyses stratified for the two age groups revealed that L-PZQ ODT without water was significantly more palatable than Rac-PZQ ODT without water in children aged between 9 and 11 years. These results, therefore, provide some evidence for the better palatability of L-PZQ ODT without water over Rac-PZQ ODT without water in older children. This was not observed in younger children. The age differences remain an important factor to accurately report on the palatability of each preparation without water.

When tasted with water, both ODT formulations were more palatable than the current PZQ-Cesol crushed in water. Like without water, the mean VAS scores of the ODT formulations were in the “Neither good nor bad” range while the PZQ-Cesol mean VAS scores were in the “Poor Taste” part of the hedonic VAS scale. Palatability reported immediately after spitting out the IMP by boys differed from what was reported by girls; boys reported higher for L-PZQ ODT and lower for PZQ-Cesol. Although limited to relatively small sample sizes of 24 boys and 23 girls, analyses stratified by gender still revealed significantly higher scores for both the new formulations dispersed in water compared with the current PZQ-Cesol formulation. It is of note that L-PZQ ODT dispersed in water was more palatable compared with Rac-PZQ ODT dispersed in water at both time points for boys but not for girls. Thus, gender continues to be an important factor along with age to consider when reporting palatability. Another observation was the effect size for the difference between two ODTs formulations was a bit fairly large and the P-value was about 0.1 (Table 3) which likely underpowered detection of the difference. Nevertheless, These findings are very important for the development of the chosen drug which has already shown promising efficacy and tolerability, which could be given as a dispersible tablet in the mouth or dispersed in water[34]

This study was limited to children aged 6 years and older; pre-school children [34] were not included as they are often shy, reluctant to participate and unable to communicate their feelings and preferences[38] However, it is reasonable to assume that the results can be extrapolated to the younger age group, who are commonly more sensitive to bitter-tasting drugs compared with older children and adults [28]. Although some parents were not familiar with the drug and the trial process before the trial started, the trial execution was a success. Our results support further development of the ODT formulations. A Phase 2 clinical study will be conducted in the targeted age group with the 2 new formulations and the one with the optimal benefit risk profile will be selected for the phase 3 pivotal study. After successful registration, a solid access pathway needs to be put in place, to allow efficient delivery and monitoring of the drug into current healthcare setups to meet the need for African children and other children across the endemic world to have better access to medication [9]. Effective early treatment is possible, thereby preventing the substantial immune-mediated effects of schistosomiasis infection[7,36]

## Conclusion

In conclusion, this trial has shown that the overall palatability of both L-PZQ ODT and Rac-PZQ ODT was higher than that of standard PZQ-Cesol when the drugs were dispersed in water in this study population of African children. The superiority of L-PZQ ODT over Rac-PZQ ODT without water could not be confirmed; however, L-PZQ ODT dispersed in water was more palatable than Rac-PZQ ODT dispersed in water. Some evidence supporting an age effect and gender effect on palatability was also observed.

## Supporting information

S1 File Taste study Protocol. (PDF)

S2 File Consort checklist Praziquantel taste study. (DOC)
