# Supplementary material for: Comparative palatability of orally disintegrating tablets (ODTs) of Praziquantel (L-PZQ and Rac-PZQ) versus current PZQ tablet in African children: A randomized, single-blind, crossover study
Source: PLoS Negl Trop Dis. 2021 Jun 9;15(6):e0007370. doi: 10.1371/journal.pntd.0007370 (PMC8216518; doi:10.1371/journal.pntd.0007370)
Supplement: S1 File — (PDF) [file pntd.0007370.s001.pdf]

Clinical Trial Protocol

**Clinical Trial Protocol Number**	EMR200661-002
**Title**	Randomized, single blind, cross-over study to evaluate the palatability of new orally disintegrating tablets of PZQ and L-PZQ versus current PZQ tablets in African children age 6-11 years.
**Phase**	Taste study (Phase I)
**Principal Investigator**	Abdunoor Mulokozi Kabanywanyi MD, PhD<br>Ifakara Health Institute, Dar es Salaam, Tanzania<br>Kibiti/Ikwiriri Rufiji Tanzania
**Sponsor**	Merck KGaA<br>Frankfurter Strasse 250<br>64293 Darmstadt, Germany
**Medical Responsible:**	Dr. Oezkan Yalkinoglu<br>Merck KGaA<br>Frankfurter Strasse 250<br>64293 Darmstadt, Germany.<br>Phone: +49 6151 72 29136<br>Fax: +49 6151 72 3163<br>Email: oezkan.yalkinoglu@merckgroup.com
**Clinical Trial Protocol Version**	02.12.14 / V4.0
**Replaces Version**	Not applicable

- Confidential –

This document is the property of Merck KGaA, Darmstadt, Germany, or one of its subsidiaries.. It is intended for restricted use only and may not – in full or part – be passed on, reproduced, published or used without express permission of Merck KGaA, Darmstadt, Germany, or its subsidiary. Copyright © 2014 by Merck KGaA, Darmstadt, Germany, or its subsidiary. All rights reserved.

Protocol Table of Contents

Protocol Table of Contents 2

Table of In-Text Tables 5

List of Abbreviations 6

1 Synopsis...7

2 Sponsor, Investigators and Trial Administrative Structure 11

3 Background Information...11

4 Trial Objectives 13

4.1 Secondary Objectives 13

5 Investigational Plan 13

5.1 Overall Trial Design and Plan 13

5.2 Discussion of Trial Design 15

5.2.1 Inclusion of Special Populations 15

5.3 Selection of Trial Population..15

5.3.1 Inclusion Criteria 16

5.3.2 Exclusion Criteria 16

5.4 Withdrawal from Trial...16

5.5 Premature Termination of the Trial 17

5.6 Definition of End of Trial..17

6 Investigational Medicinal Product(s) and Other Drugs Used in the Trial...18

6.1 Description of Investigational Medicinal Product(s)..........................18

6.2 Dosage and Administration 19

6.3 Assignment to Treatment Groups.......................................20

6.4 Other Drugs to be used in the Trial 21

6.5 Concomitant Medications and Therapies 21

6.5.1 Permitted Medicines 21

6.5.2 Non-permitted Medicines 22

6.5.3 Other Trial Considerations 22

6.5.4 Special Precautions...22

6.5.5 Management of Specific Adverse Events or Adverse Drug Reactions...23

6.6 Packaging and Labeling....23
6.7 Preparation, Handling and Storage....23
6.8 Investigational Medicinal Product Accountability....24
6.9 Assessment of Investigational Medicinal Product Compliance....24
6.10 Method of Blinding....25
6.11 Emergency Unblinding....25
6.12 Treatment of Overdose....25
6.13 Medical Care of Subjects after End of Trial....25
7 Trial Procedures and Assessments....26
7.1 Schedule of assessments....26
7.1.1 Screening procedures....26
7.1.2 Study Day 1....26
7.1.3 Study Day 2....27
7.1.4 Post study....28
7.1.5 Schedule of Assessments....29
7.2 Demographic and Other Baseline Characteristics....31
7.3 Palatability Assessments....31
7.4 Assessment of Safety....31
7.4.1 Adverse Events....32
7.4.2 Vital Signs, Physical Examinations, and Other Assessments....36
8 Statistics....36
8.1 Sample Size....36
8.2 Randomization....37
8.3 Endpoints....37
8.3.1 Primary Endpoints....37
8.3.2 Secondary Endpoints....37
8.4 Analysis Sets....38
8.4.1 Full Analysis Set....38
8.4.2 Safety Set....38
8.4.3 Completers Analysis Set....38
8.5 Description of Statistical Analyses....38
8.5.1 General Considerations....38

8.5.2 Analysis of Primary Endpoints ... 39
8.5.3 Analysis of Secondary Endpoints ... 39
8.5.4 Analysis of Safety and Other Endpoints ... 39
9 Ethical and Regulatory Aspects ... 40
9.1 Responsibilities of the Investigator ... 40
9.2 Subject Information and Informed Consent ... 40
9.3 Subject Identification and Privacy ... 41
9.4 Emergency Medical Support and Subject Card ... 42
9.5 Clinical Trial Insurance and Compensation to Subjects ... 42
9.6 Independent Ethics Committee or Institutional Review Board ... 42
9.7 Health Authorities ... 43
10 Trial Management ... 43
10.1 Case Report Form Handling ... 43
10.2 Source Data and Subject Files ... 43
10.3 Investigator Site File and Archiving ... 44
10.4 Monitoring, Quality Assurance and Inspection by Health Authorities ... 44
10.5 Changes to the Clinical Trial Protocol ... 45
10.6 Clinical Trial Report and Publication Policy ... 45
10.6.1 Clinical Trial Report ... 45
10.6.2 Publication ... 45
Appendix I: Signature Pages and Responsible Persons for the Trial ... 46
Signature Page – Protocol Lead ... 47
Signature Page – Principal Investigator ... 48
Sponsor Responsible Person ... 49
Appendix II: References ... 50
Appendix III: Questionnaire ... 51

Table of In-Text Tables

Figure 1- Schedule of assessment 10
Figure 2- Schedule of assessment (detailed timing) 30

List of Abbreviations

AE	Adverse Event
API	Active Pharmaceutical Ingredient
CRF	Case Report Form
CHMP	Committee for medical product for human use
ECG	Electrocardiogram
EMA	European Medicines Agency
FDA	Food and Drug Administration
GCP	Good Clinical Practice
IB	Investigator’s Brochure
ICH	International Conference on Harmonization
IEC	Independent Ethics Committee
IMP	Investigational Medicinal Product
IRB	Institutional Review Board
ITT	Intention To Treat
NIMR	National Institute for Medical Research, Tanzania
NTD	Neglected Tropical Disease
ODT	Orally disintegrating tablets
PACTR	Pan African Clinical Trials Registry
PD	Pharmacodynamics
PGx	Pharmacogenetics/Pharmacogenomics
PK	Pharmacokinetics
Pre-SAC	pre-School Aged Children
PZQ	Praziquantel
Rac	Racemate
SAE	Serious Adverse Event
SMC	Safety Monitoring Committee
STH	Soil Transmitted Helminthes
VAS	Visual Analogue Scale
WHA	Word Health Agency
WHO	Word Health Organization

1 Synopsis

**Clinical Trial Protocol Number**	EMR200661-002
**Title**	Randomized, single blind, cross-over study to evaluate the palatability of new orally disintegrating tablets of PZQ and L-PZQ versus current PZQ tablets in African children age 6-11 years.
**Trial Phase**	Taste study (Pre-phase I)
**IND Number**	Not applicable
**FDA covered trial**	No
**Principal Investigator**	Abdunoor Mulokozi
**Sponsor**	Merck KGaA<br>Frankfurter Strasse 250<br>64293 Darmstadt
**Trial centers/countries**	The study will be conducted in a primary school in Tanzania.<br>Previous PZQ treatments and schistosomiasis prevalence in the area will be recorded.
**Planned trial period (first subject in-last subject out)**	Enrolment start date (FPI): Q1 2015<br>Enrolment finish date (LPI): Q1 2015
**Trial Registry**	Clintrials.gov, PACTR

Objectives:

Primary objective

The primary objective of the trial is to compare the “overall palatability” of the new L-PZQ ODTs, the new racemate PZQ ODTs (Rac-PZQ ODT) and the current available racemate PZQ tablets (reference) as assessed by means of human gustatory sensation tests (100 mm Visual analogue scale (VAS) scoring modified by the incorporation of a 5 point facial hedonic scale).

Secondary objectives

- To obtain feedback from these children regarding taste of the different formulations using an open ended questionnaire.
- To document any discomfort or other observation in relation to acceptance of the study medication

Methodology: Initially pupils will be invited with their parents to school for screening whereas they will be asked to consent/assent in to the study and they will be instructed on how to follow up the taste study procedures. Enrolments will occur in the health facilities in Ikwiriri/Kibiti at the end of successive training. Informed consent from parents and guardians and assent from the child will be obtained before participation in the study. The study will be

conducted in school children 6 years and older as recommended by the CHMP (Committee for medical product for human use) reflection paper: formulations of choice for the pediatric population).

- This is a randomized, five-period cross over, single center swill and spit taste study where the drug will not be swallowed but will be spit out after tasting.

On Day 1, the subjects will assess the palatability of the following arms in a randomized sequence:

- L-PZQ ODT (150 mg) put and disintegrated in the mouth
- Rac- PZQ ODT (150 mg) put and disintegrated in the mouth

On Day 2, the subjects will assess the palatability of the following arms in a randomized sequence:

- L-PZQ ODT (150 mg) dispersed in water administered in the mouth cavity
- Rac- ODT (150 mg) dispersed in water administered in the mouth cavity
- 150 mg current PZQ tablet (1/4 of a 600 mg tablet) crushed, dispersed in water and administered in the mouth cavity

Gustatory sensation studies will be performed on the different formulations immediately after tasting and 2-5 min after the study drug has been spat out.

All volunteers will be asked to place a mark along the line with the use of a 100 mm visual analogue scale (VAS) that incorporates a 5 points hedonic scale for "overall palatability".

In addition, any discomfort or other observation in relation to acceptance of the study medication (e. g. spitting out of the medicine) will be reported by the parents or investigator.

An open ended questionnaire (description of mouth feeling and taste description) will be conducted for each child during the washout period.

After the trial a therapeutic dose of Praziquantel will be made available to the local health council and management team to provide to the participating school.

Planned number of subjects: 48 healthy children (1:1 female/male ratio) will be randomized Children age 6-8 and 9-11 will be stratified in 2 equal groups to have a representative number of children across the full age range 6-11 years.

Primary endpoints: Primary endpoints are results from Visual Analogue score (VAS score) assessments (100 mm VAS scale modified by the incorporation of a 5 point facial hedonic scale). Primary endpoint point for the taste is VAS taken at 0 min.

The data will be evaluated using ANOVA with time, sequence, treatment and stratification factors (age and sex as covariates)

Secondary endpoints:

- Results from VAS taken at 2-5 min

- Description of mouth feeling and taste description using an open ended questionnaire

- Any discomfort or other observation in relation to acceptance of the study drug will be recorded in the CRF

Diagnosis and key inclusion and exclusion criteria:

For inclusion in the trial, all of the following **inclusion criteria** must be met:<br>1. Children male or female aged 6-11 years (inclusive).<br>2. Parents or guardians gave written informed consent prior to any trial related procedure and child gave assent<br>3. Able to communicate well with the Investigator, understanding the protocol requirements and restrictions, and willing to comply with the requirements of the entire trial.<br>4. Subjects should be able to hold a small volume of any appropriate juice in their mouth for 10 sec., without swallowing it and to keep a candy in the mouth for 20 sec. without swallowing it.<br>5. Children who are able to properly assess and differentiate flavours of different soft drinks.<br>6. Children who are able to use a hedonic scale (children will be trained before the study).<br><br>Subjects are not eligible for this trial if they meet any of the following **exclusion criteria**:<br>1. Unlikely to comply with the protocol requirements, instructions and trial-related restrictions, e.g., uncooperative attitude, inability to return for follow-up visits, and improbability of completing the trial.<br>2. Children with any condition or dietary habit known to interfere with the sense of smell and taste, ingestion of any medication (except paracetamol)<br>3. Children with significant illness in the previous 2 weeks<br>4. Any surgical or medical condition, or any significant disease that in the opinion of the investigator, constitutes a risk or a contraindication for the participation of the subject in the study that could interfere with the study objectives, conduct or evaluation.<br>5. Children who have participated in any clinical investigation within the previous 4 weeks.
**Investigational Medicinal Product:**<br>L- PZQ 150 mg orally disintegrating tablets (ODTs)<br>Racemate- PZQ 150 mg orally disintegrating tablets (ODTs)<br>**Mode of administration:** In the oral cavity but the drug will not be swallowed<br>The ODTs will be either dispersed in water (C, D) or put directly on the tongue (A and B).
**Reference IMP: dose/mode of administration/dosing schedule:**<br>Current PZQ tablets (Cesol 600 mg)<br>**Mode of administration:** The tablet will be crushed and dispersed in water and ¼ of this dispersion (containing 150 mg), will be administered in the mouth cavity but not swallowed. .
**Planned trial and treatment duration per subject:** One treatment period of 2 days and a screening period of up to 14 days results in up to 3 weeks per subject.

The study is a two period, single blind cross-over design on Day 1 and a three period single blind cross-over on Day 2. The palatability assessments on Day 1 will involve the auto-disintegration of ODTs that will be put and disperse themselves in the mouth cavity to be followed by spit of the dispersed tablet. On Day 2, the swill and spit of the solutions made from both tablets in water

will take place. In addition to the tasting of the Rac-PZQ ODT and L-PZQ ODT on Day 2, the current Praziquantel tablets will be crushed and then dispersed in water for similar tasting & spitting thereof. The schedule of assessment is detailed in Table 1 below.

Table 1 Schedule of Assessments

DAY 1		
PERIOD 1	PERIOD 2	
A	B	
B	A	
DAY 2		
PERIOD 3	PERIOD 4	PERIOD 5
C	D	E
C	E	D
D	E	C
D	C	E
E	C	D
E	D	C

A) L-PZQ ODT put and disintegrated in the mouth without water
B) Rac-PZQ ODT put and disintegrated in the mouth without water.
C) L-PZQ ODT dispersed in water.
D) Rac-PZQ ODT dispersed in water
E) Current PZQ formulation 150 mg crushed in water

2 Sponsor, Investigators and Trial Administrative Structure

This clinical trial will be sponsored by:

Merck KGaA

Frankfurter Strasse 250

64293 Darmstadt

The trial will be conducted at one site of the Ifakara Health Institute (Rufiji branch) – Tanzania

The Principal Investigator Abdunoor Mulokozi, Ifakara Health Institute, represents all Investigators for decisions and discussions regarding this trial, consistent with the International Conference on Harmonisation (ICH) Topic E6 Good Clinical Practice (GCP; hereafter referred to as ICH GCP). The Principal Investigator will provide expert medical input and advice relating to trial design and execution and is responsible for the review and signoff of the clinical trial report. The PI has experience in conducting Taste studies.

Signature pages for the Protocol Lead and the Principal Investigator as well as a list of Sponsor responsible persons are in Appendix I.

The trial will appear in the following clinical trial registries: clinicaltrials.gov, Pan African Clinical Trials Registry (PACTR).

The trial will be fully outsourced including monitoring, to the Swiss Tropical Health Institute, (Swiss TPH), one of the Praziquantel Pediatric consortium partners.

The study medication will be provided by the sponsor directly to the trial site.

3 Background Information

Schistosomiasis, also called Bilharzia, belongs to one of the most neglected tropical diseases caused by flatworms which remain one of the most prevalent parasitic diseases in developing countries. After malaria, schistosomiasis is the most important tropical disease in terms of human morbidity with significant economic and public health consequences. The disease is a severe chronic inflammatory disease and is endemic in about 75 developing countries, infecting more than 220 million people, with more than 90% of them living in Africa. They live in rural agricultural and peri-urban areas, and placing more than 700 million people at risk. Of the infected patients, 20 million suffer severe consequences from the disease. Some estimate that there are approximately 20,000 deaths related to schistosomiasis yearly. In many areas, schistosomiasis infects a large proportion of children under the age of 14 years.

The WHO recommended control strategy of schistosomiasis is based on preventive chemotherapy interventions targeting the majority of the at-risk population. The current gold standard treatment employs annual single oral dose of the drug Praziquantel (PZQ) 600 mg tablets, jointly developed by Bayer-Merck in the 1970's, and commercialized as Biltricide

(Bayer), and Cesol/Cisticide 500/600 mg (Merck KGaA) for human use, as well as other generic products.

The prevalence of schistosomiasis in children is very high, accounting for about 50% of the total infected population of 220 million and many more are at risk from the disease. At the World Health Assembly (WHA) in 2001, Resolution A 54.19 was put forward which urged endemic countries to start seriously tackling worms, specifically schistosomiasis and soil transmitted helminthes (STH), with a global target to treat at least 75% of all school aged children who are at risk of morbidity from schistosomiasis and STH by the year 2010. However, pre-school aged children (pre-SAC), which are a high-risk group for schistosome infections counting for 10-20 million of the global prevalence, are currently not treated as they are not included in the schistosomiasis control programs. WHA passed Resolution 66.20 (2013) where the Member States essentially confirmed their commitment to fulfil the 2020 Roadmap for Neglected tropical diseases (NTDs).

To address the gap of non-treatment of pre-SAC children, a Praziquantel pediatric consortium has been set-up under the umbrella of Top Institute Pharma (TI Pharma, Leiden, Netherlands) with partners from the pharmaceutical industry (Merck Serono, Germany, Astellas Pharma, Japan, SimCyp, UK), the public sector (Farmanghuinos, Brazil) and the academic sector (Swiss Tropical Public and Health Institute –Swiss TPH). In this trial the Ifakara Health Institute (IHI) partners with Swiss TPH to undertake this study in Tanzania. The consortium aspires to develop a new pediatric formulation of PZQ and register its use in the pediatric schistosomiasis indication.

PZQ is a racemic mixture composed of the L-PZQ and D-PZQ enantiomers in a 1:1 ratio. The cidal activity on the worms resides in the L-PZQ enantiomer whereas the D-PZQ enantiomer was suggested to play an important role in the bitterness. Therefore, a new L-PZQ formulation (including taste masking by using mannitol and sweeteners) is expected to give better palatability (less bitterness) than a rac-PZQ formulation. Both L-PZQ and rac-PZQ child friendly oral disintegrating tablet (ODT) formulations for pediatric use are currently under development and the most suitable formulation will be selected in 2015 for further development and registration in children age 3 months to 6 years infected with Schistosomiasis.

This project has been presented and approved by the PZQ Consortium Board in Dec 2013 and the Merck Integrated Clinical Study Committee on the 08 Jun 2014. For further information about the nonclinical and clinical programs and Guidance for the Investigator, please refer to the Investigator’s Brochures (IB).

This clinical trial will be conducted in compliance with the clinical trial protocol, ICH GCP, and any additional applicable regulatory requirements.

Based on the available nonclinical and clinical data of PZQ and L-PZQ to date, the conduct of the trial specified in this protocol is considered justifiable.

4 Trial Objectives

- To compare the "overall palatability" of the new L-PZQ ODT (without water) and the new racemate PZQ ODT (without water)
- To compare the "overall palatability" of the new L-PZQ ODT (with water), the new Rac-PZQ ODT (with water) and the current racemate PZQ tablets (crushed and dispersed with water) (reference).

Assessment will be done by means of human gustatory sensation tests (100 mm VAS scale modified by the incorporation of a 5 point facial hedonic scale). Primary end point for the taste is the VAS scale taken at 0 min. The VAS scale obtained at 2 to 5 minutes is a secondary endpoint for the taste. The data will be evaluated using ANOVA with sequence, treatment and stratification factors (age and sex as covariates)

4.1 Secondary Objectives

- To obtain feedback regarding mouth feeling and taste of the different formulations
- To document any discomfort or other observation in relation to acceptance of the study medication

An open ended questionnaire will be used. Any discomfort or other observation in relation to acceptance of the study medication will be recorded in the CRF.

5 Investigational Plan

5.1 Overall Trial Design and Plan

The participating primary school has been informally contacted and further formal contacts through the Rufiji district education officer will be established upon ethics approval from MRCC. The children will be recruited from one primary school, in the Rufiji district. Initially pupils will be invited with their parents to school for screening whereas they will be asked to consent/assent in to the study and they will be instructed on how to follow up the taste study procedures. Enrolments will occur in the health facilities in Ikwiriri/Kibiti at the end of successive training.

During a two weeks screening period, children will be tested for their ability to hold 2 ml of any appropriate juice in their mouth for 10 seconds and then expel; and to keep a candy in the mouth for 20 sec. without swallowing it. In addition children will be assessed for gustatory abnormality before inclusion in to the study. This prior assessment will involve the documentation of candidate subjects' ability to properly differentiate flavours of different drinks. In addition, the children will be trained on how to use a hedonic scale.

Initial screening will happen two weeks ahead of trial test date. All pupils meeting inclusion criteria will be further trained on how to hold a test fluid in their mouth. Those exercises will occur during the up to two weeks preceding the taste study. At the end of the screening activities

subjects who meet the inclusion/exclusion criteria will be asked to return to school for the taste assessments during the weekend. Subjects along with their parents/ legal representative will be asked to report to the study site at least 2 hour prior to the first taste test.

As per study instruction children will be asked to perform a swill and spit test of the taste regimen in the form of L-PZQ ODT and Rac-PZQ ODT (each once put on the tongue or dispersed in water) and the current Praziquantel tablet dispersed in water after crushing.

In order not to stress the children and to ensure the complete washing out period of at least one hour, the 5 taste assessment periods are split over two days. Dispersible tablets will be left to self-disintegration without dissolution in water in the buccal cavity for the two tastings on Day 1 whereas the assessment of the dispersed in water taste candidates will occur on Day 2.

A 1;1 ratio of gender based random assignment will ensure equal distribution of subjects' in groups of equal age categories. Each individual will taste each preparation once making up 5 periods for each sequence of the palatability assessment during the two study days (see Table below)

According to the gender and age each subject will be randomized to one of the sequences.

DAY 1	
PERIOD 1	PERIOD 2
A	B
B	A

DAY 2		
PERIOD 3	PERIOD 4	PERIOD 5
C	D	E
C	E	D
D	E	C
D	C	E
E	C	D
E	D	C

A) L-PZQ ODT put and disintegrated in the mouth without water
B) Rac-PZQ ODT put and disintegrated in the mouth without water.
C) L-PZQ ODT dispersed in water.
D) Rac-PZQ ODT dispersed in water
E) Current PZQ formulation 150 mg crushed in water

For every taste, immediately after spitting and 2-5 min thereafter, the child will be asked to put a mark along the line of the 100 mm visual analogue scale (VAS) incorporating a 5 points hedonic scale. An open ended questionnaire (description of mouth feeling and taste description) will be conducted. A detailed description of the assessment is available in section 7/Appendix III.

After the trial a therapeutic dose of Praziquantel will be made available to the local health council and management team to provide to the participating school.

5.2 Discussion of Trial Design

The trial will enrol infected and non-infected children, and diagnosis of schistosomiasis will not be made and is not part of the inclusion criteria. According to the EU guidance “Ethical considerations for clinical trials on medicinal products conducted with the pediatric population” it is acceptable to conduct swill and spit taste study in healthy children. However, following the Advisory Board Expert meeting held in August 2013 in Geneva, inclusion of infected children was deemed appropriate and it was recommended to provide a prophylactic post-medication therapeutic dose treatment of Praziquantel to all children who have participated to the trial after the study ends (according to WHO SOPs). It was also recommended at this Expert meeting to avoid exposing children to unnecessary taste fatigue (due to the unpleasant taste of PZQ and the number of treatment arms), as recommended in the CHMP guidance (CHMP, 2005) and to limit as much as possible the number of tablets of PZQ per treatment arm. Hence, a single tablet (150 mg L-PZQ ODT, 150 mg rac-PZQ ODT or ¼ of the Cesol 600 mg tablet) is administered in the trial. To avoid taste fatigue the 5 taste assessments have been spread over two days.

5.2.1 Inclusion of Special Populations

African children will be included in this study.

Children are not small adults and children as the target population is regarded as the most suitable panel for taste assessment of pediatric population.

It is recommended to use children over 6 as children below 4-6 years are not considered to be able to express differences in taste perception.

According to the EU guidance “Ethical considerations for clinical trials on medicinal products conducted with the pediatric population” it is acceptable to conduct swill and spit taste study in healthy children. Therefore, healthy children are also included in the trial (see section 5.2.2,, inclusion criteria).

There is only a positive benefit /risk for African children since they will have the possibility to receive a therapeutic dose of PZQ after the study

5.3 Selection of Trial Population

Only persons meeting all inclusion criteria and not meeting any of the exclusion criteria may be enrolled into the trial as subjects. Prior to performing any trial assessments not part of the subject’s routine medical care, the Investigator will ensure that the subject’s legal representative has provided written informed consent following the procedure described in Section 9.2. and that the subject has given assent.

5.3.1 Inclusion Criteria

For inclusion in the study, all of the following inclusion criteria must be fulfilled:

1. Children, male or female aged 6-11 years (included). Age will be documented by verifying date of birth available from school admission registries or birth certificates in case they are readily available.
2. Parents or guardians given written informed consent prior to any trial related procedure and child given assent.
3. Able to communicate well with the Investigator, understanding the protocol requirements and restrictions, and willing to comply with the requirements of the entire trial.
4. Subjects should be able to hold 2 ml of any appropriate juice in their mouth for 10 sec., without swallowing it and to keep a candy in the mouth for 20 sec. without swallowing it.
5. Children who are able to properly assess and differentiate flavours of different soft drinks.
6. Children who are able to use a haedonic scale (children will be trained before the study).

5.3.2 Exclusion Criteria

Subjects are not eligible for this study if they fulfil one or more of the following exclusion criteria:

7. Unlikely to comply with the protocol requirements, instructions and trial-related restrictions, e.g., uncooperative attitude, inability to return for follow-up visits, and improbability of completing the trial.
8. Children with any condition or dietary habit known to interfere with the sense of smell and taste, ingestion of any medication (except paracetamol)
9. All febrile children or those found with history of any significant illness in the period of two weeks preceding the trial. Significant illness among school children attending classes is defined as presence of high body temperature >=38°C, respiratory and heart rates above normal ranges based on the opinion of the study clinician and presence of severe oral thrush.. Those identified sick children at screening will be referred to clinics for management.
10. Any surgical or medical condition, including findings in the medical history, or any significant disease (including children with history of autonomic dysfunction, bronchospastic disease, known hypersensitivity to any drug or artificial sweetener) that in the opinion of the investigator, constitutes a risk or a contraindication for the participation of the subject in the study or that could interfere with the study objectives, conduct or evaluation.
11. Children who have participated in any clinical investigation within the previous 4 weeks.

5.4 Withdrawal from Trial

Subjects can be withdrawn from the study procedures for the following reasons:

- Any serious adverse event (SAE).
- Subject lost to follow-up after several documented attempts to contact them.
- Subject withdrew consent.
- Any events that unacceptably endanger the safety of the subject.
- Subject who inadvertently swallow the medication during more than 1 taste assessment.

If a volunteer fails to appear for Day 2 or cannot complete the 5 taste assessments, efforts (i.e. documented phone calls to parents) will be undertaken to locate or recall the subject or at least to determine his/her health status and the explanation for early termination. Those volunteers that cannot complete 5 taste assessments will be replaced by a volunteer of the same sex and age group.

These efforts will be documented in the volunteer's CRF and source documents.

5.5 Premature Termination of the Trial

The clinical trial may be terminated prematurely or suspended at the request of Health Authorities or if new safety or efficacy information leads to an unfavourable risk benefit judgment for any IMP. The Sponsor may discontinue the trial if it becomes unjustifiable for medical or ethical reasons, for poor enrolment, or because of discontinuation of clinical development of an IMP or withdrawal of an IMP or comparator from the market for safety reasons.

Health Authorities and Independent Ethics Committees (IECs)/Institutional Review Boards (IRBs) will be informed about the discontinuation of the trial in accordance with applicable regulations.

5.6 Definition of End of Trial

End-of-study evaluations will be performed at end of tasting period. End-of-study evaluations must also be performed when a subject prematurely terminates the study.

A clinical trial protocol may not be considered closed as long as:

- Any subject is still receiving any IMP,
- Visits specified by the protocol are still taking place,
- Procedures or interventions according to the protocol are still being undertaken in any subject,
- The post-treatment follow up period, defined in the clinical trial protocol as being part of the trial, has not yet been completed for any subject.

6 Investigational Medicinal Product(s) and Other Drugs Used in the Trial

6.1 Description of Investigational Medicinal Product(s)

MSC 2499550A (referred to as L-PZQ)

Chemical Name: (11bR)-2-(Cyclohexylcarbonyl)-1,2,3,6,7,11b-hexahydro-4H-pyrazino[2,1-a]isoquinolin-4-one

Recommended International Nonproprietary name (rINN): R-(-)-Praziquantel

Company or Laboratory Code: MSC2499550A

Molecular Formula: $C_{19}H_{24}N_2O_2$

Relative Molecular Mass: 312.4 g/mol

White or almost white, crystalline powder.

MSC2499550A is presented as 150 mg strength ODT.

The tablet consists of MSC2499550A combined with the following pharmaceutical grade excipients: Maize starch, mannitol, sucralose, magnesium stearate and colloidal silicon dioxide.

MSC 1028703A (referred to as rac-PZQ)

Chemical Name: (11bRS)-2-(Cyclohexylcarbonyl)-1,2,3,6,7,11b-hexahydro-4H-pyrazino[2,1-a]isoquinolin-4-one

Recommended International Nonproprietary name (rINN): (RS)-Praziquantel

Company or Laboratory Code: MSC1028703A

Molecular Formula: $C_{19}H_{24}N_2O_2$

Relative Molecular Mass: 312.4 g/mol

White or almost white, crystalline powder.

MSC1028703A is presented as 150 mg strength ODT.

The tablet consists of MSC1028703A combined with the following pharmaceutical grade excipients: starch 1500, mannitol, sucralose, magnesium stearate and colloidal silicon dioxide.

Praziquantel (Cesol® 600 mg)

Chemical Name: (11bRS)-2-(Cyclohexylcarbonyl)-1,2,3,6,7,11b-hexahydro-4H-pyrazino[2,1-a]isoquinolin-4-one

Recommended International Nonproprietary name (rINN): (RS)-Praziquantel

Molecular Formula: $C_{19}H_{24}N_2O_2$

Relative Molecular Mass: 312.4 g/mol

White oblong tablet with one dividing score.

The tablet, which is available in Tanzania through the WHO donation program, consists of pharmaceutical grade excipients: sodium lauryl sulfate, magnesium stearate, corn starch, microcrystalline cellulose and povidone.

6.2 Dosage and Administration

The dosages to be administered in this trial are a single 150 mg ODT of either L-PZQ ODT tablet (MSC2499550A) or Rac-PZQ ODT tablet (MSC1028703A) or ¼ tablet dose of Cesol® 600 mg.

The tasting will last 2 days for each subject. On each day, the test will start in the morning after breakfast. The composition of the breakfast will be documented as it will be offered to the study participants by the study team and therefore it will be the same for each subject.

For Treatments arms A and B, the L-PZQ (MSC2499550A) ODT tablet or the Rac-PZQ (MSC1028703A) ODT tablet respectively will be placed directly on the tongue of the child (without water). Then the child will be asked to spit out the tablet in a waste container followed by rinsing with water to ensure all particles have been cleared off the mouth. A mouth check should be performed after the assessment to ensure that no particles remain in the oral cavity. If any IMP particles are visible the child should be asked to re-rinse its mouth with water and a recheck is performed. For treatment arms C and D, the L-PZQ (MSC2499550A) ODT tablet or the rac-PZQ (MSC1028703A) ODT tablet respectively will be dispersed in water (without gas), swirled, and about 150 mg dose of the medication administered in the buccal cavity using a syringe. The child will be asked to hold the solution for 10 sec in its mouth and will then spit out the liquid in a waste container. Rinsing with water to ensure all particles have been cleared off the mouth will be done after every taste test. A mouth check should be performed after the administration to ensure that no particles remain in the oral cavity. If any IMP particles are visible the child should be asked to rinse the mouth again with water and a recheck is performed.

For treatment arm 5, PZQ tablets (Cesol®) will be crushed and suspended in water and ¼ of the suspension (around 150 mg) will be administered directly in the buccal cavity using a syringe.

The child will be asked to hold the suspension for 10 sec in its mouth and will then spit out the liquid in a waste container. Rinsing with water to ensure all particles have been cleared off the mouth will be done after the spiting of the liquid. A mouth check will be performed after rinsing to ensure that no particles remain in the oral cavity. If any IMP particles are visible the child should be asked to rinse its mouth again with water and a recheck is performed.

For all treatments arms detailed preparation and administration instructions will be available in a separate document before the start of the study.

6.3 Assignment to Treatment Groups

Subjects will receive their subject identification number after the parents/guardians have signed their informed consent and the subject their assent. Subjects who meet all inclusion criteria and none of the exclusion criteria will be randomized after recheck of eligibility on Day 1.

The Investigator or delegate will allocate a randomization number to each subject in each stratification group, in sequential order before first tasting of IMP.

48 children (1:1 female/male ratio) will be included in the study.
The children will be randomized in blocks of gender and age (6 to <9 year old and >=9 to <12 years old) in the order in which they are enrolled according to a randomization schedule prepared by Swiss TPH. Sequential randomly assigned numbers will be kept in sealed envelopes for each individual subject.
After signature of the informed consent and assent and the inclusion/exclusion criteria have been confirmed, the Investigator will open the next envelope corresponding to the correct gender and age group to assign the sequence in which the child will have to assess the tastes of the different treatments.

The randomization code consists of a single letter for the sequence in Day 1 and a letter for Day 2 is provided in table 2.
1 stands for administered by putting on the tongue (always done on Day 1)
2 stands for administered by dispersing/crushing in water (always done on Day 2)

Table 2 Randomization codes and sequences

**Randomization code**	**DAY 1**	
F1	Rac-PZQ ODT put on the tongue (B)	L-PZQ ODT put on the tongue (A)
G1	L-PZQ ODT put on the tongue (A)	Rac--PZQ ODT put on the tongue (B)
	**DAY 2**	

H2	L-PZQ ODT dispersed in water (C)	Rac-PZQ ODT dispersed in water (D)	PZQ formulation 150 mg crushed in water (E)
J2	L-PZQ ODT dispersed in water (C)	PZQ formulation 150 mg crushed in water (E)	Rac-PZQ ODT dispersed in water (D)
K2	Rac-PZQ ODT dispersed in water (D)	PZQ formulation 150 mg crushed in water (E)	L-PZQ ODT dispersed in water (C)
L2	Rac-PZQ ODT dispersed in water (D)	L-PZQ ODT dispersed in water (C)	PZQ formulation 150 mg crushed in water (E)
M2	PZQ formulation 150 mg crushed in water (E)	L-PZQ ODT dispersed in water (C)	Rac-PZQ ODT dispersed in water (D)
N2	PZQ formulation 150 mg crushed in water (E)	Rac-PZQ ODT dispersed in water (D)	L-PZQ ODT dispersed in water (C)

The pharmacist will perform the randomization by taking the next envelope corresponding to the child's gender and age.

In order to avoid assignment errors the envelopes for boys and girls will be of different colour and the envelopes for the two age groups will also differ (e.g. blue envelopes for boy and age group 6-9 years has a different sticker than age group 9-11). In the randomization envelope there will be the code for the sequence of taste assessment for all 5 treatments (i.e. for both days).

If a volunteer does not show up on Day 2, swallows more than 1 medication or cannot assess all 5 taste assessments he/she will be replaced by a volunteer of the same sex and age group.

6.4 Other Drugs to be used in the Trial

Not applicable.

6.5 Concomitant Medications and Therapies

6.5.1 Permitted Medicines

Any medications (other than those excluded by the clinical trial protocol) that are considered necessary for the subjects' welfare and will not interfere with the trial medication may be given at the Investigator's discretion.

The Investigator will record all concomitant medications taken by the subject during the trial, from the date of signature of informed consent, in the appropriate section of the CRF.

Any additional concomitant therapy that becomes necessary during the trial and any change to concomitant drugs must be recorded in the corresponding section of the CRF, noting the name, dose, duration and indication of each drug.

6.5.2 Non-permitted Medicines

Administration of any investigational product or use of any investigational device 4 weeks prior to first administration of IMP and during the entire clinical trial is not permitted.

Also, any medication from screening until last administration is not allowed without prior approval from the Investigator. No vitamins, supplements or herbal remedies are allowed. Attention should be paid to any drugs or OTC compounds that may affect the taste study assessment.

Any additional concomitant therapy that becomes necessary during the trial must be recorded in the corresponding section of the CRF, noting the name, dose, duration and indication of each drug.

If the administration of a non-permitted concomitant drug becomes necessary during the trial, e.g., because of AEs, the subject should be discontinued from the trial but only after consultation with the Sponsor.

6.5.3 Other Trial Considerations

Any unplanned diagnostic, therapeutic, or surgical procedure performed during the trial period must be recorded in the concomitant procedure section in the CRF, including the date, indication, and description of the procedure(s) and outcome.

Any other special considerations (for example, concerning food, caffeine and physical exercise) should be noted.

Subjects must have been fasting for at least 1 hr before IMP tasting. Five minutes prior to each tasting, the subjects will cleanse the palate by eating one cracker and drinking a small cup of room temperature drinking water (without gas). The subjects will be instructed not to swallow the study medication. The study personnel will confirm that no study drug was swallowed by checking the container into which the study drug was expelled.

Following the evaluation and completion of the questionnaire, approximately 5 minutes later, the subjects will rinse the mouth with drinking a small cup of water and eating a cracker, to prepare for the next test. Subsequent tasting tests will occur after an interval of at about 60 min.

Subjects will be instructed not to consume caffeine or xanthine-containing products (chocolate, tea, coffee, cola, energy drinks, etc.) from 48 hours prior to the first administration until the end of the trial.

6.5.4 Special Precautions

Subjects will be recruited from one school, in Rufiji district, Tanzania in Africa. Participants will be invited to the neighbouring public health facility serving the area where the trial will be performed in a clinic with access to basic emergency facilities. Equipment and other agents

(epinephrine, prednisolone equivalents, etc.) will be available at the trial site in case of severe allergic reactions.

6.5.5 Management of Specific Adverse Events or Adverse Drug Reactions

As this is a Swill and Spit study, no safety or tolerability concerns are expected (drug not swallowed). Even in case of inadvertent swallowing of a 150 mg tablet or in case of unexpected high buccal absorption, safety concerns should not be raised since the 150 mg ODT dose is under the used therapeutic dose of 40 mg/kg rac-PZQ (7 rac-PZQ ODTs or 3 L-PZQ ODTs needed to treat a 25 kg 6 year old child).

Children are kept under observation for least 2 hour after the last taste assessment before leaving the unit. Spontaneously reported adverse events (by parents/guardians) will be managed in the participating health facility by study medical doctor who will remain in the facility during the trial and two weeks thereafter. AEs will be recorded in the CRF until one week after the last taste assessment.

6.6 Packaging and Labelling

Primary packaging and labelling of IMP will be done by the sponsor in accordance with applicable local regulatory requirements and applicable Good Manufacturing Practice (GMP) Guidelines.

Packaging and labelling of MSC2499550A and MSC1028703A ODTs will be described in detail in the Packaging Concept. Packaging units will be numbered. One packaging unit will be used for multiple subjects. In addition retention samples will be provided as required by guidelines.

Current PZQ (Cesol 600 mg®) is packed at 1000 tablets per bottle. Only one bottle will be used. Labelling of the bottle will be detailed in the Packaging Concept.

Other accessories (e.g. syringes, beakers [disposable]) for dispensing study drug will be provided as bulk supply.

6.7 Preparation, Handling and Storage

The preparation of the dispersed or reconstituted solution for the individual subject (for treatment arms C, D, E) will be done by a competent person (e.g., a pharmacist) under the responsibility of the Investigator. The pharmacy will prepare all IMPs for all subjects per period according to the treatment randomization list, and in accordance with the Pharmacy Manual.

MSC2499550A, MSC1028703A and Cesol® shall be stored protected from light below or at 30°C in a secure, locked location with adequate storage conditions. Any deviations from the recommended storage conditions should be immediately reported to the Sponsor and the IMPs should not be used until authorization has been received from the Sponsor.

6.8 Investigational Medicinal Product Accountability

The Investigator is responsible for ensuring accountability for IMP, including reconciliation of drugs and maintenance of drug records.

- Upon receipt of IMP, the Investigator (or designee) will check for accurate delivery and acknowledge receipt by signing (or initialing) and dating the documentation provided by the Sponsor/CMO and returning it to the Sponsor/CMO. A copy will be retained for the Investigator File.
- The dispensing of the IMP will be carefully recorded on the appropriate drug accountability forms and an accurate accounting will be available for verification by the Sponsor Monitor at each monitoring visit.
- IMP accountability records will include:
  - Confirmation of IMP delivery to the trial site in good condition and within the defined temperature range.
  - The inventory at the site of IMP provided by the Sponsor/CMO and prepared at the site.
  - The use of each dose by each subject.
  - The disposition of unused IMP.
  - Dates, quantities, batch numbers, expiry dates and (for IMP prepared at the site) formulation, as well as the subjects' trial numbers.
- The Investigator should maintain records that adequately document:
  - That the subjects were provided the doses specified by the clinical trial protocol/amendment(s), and
  - That all IMP provided by the Sponsor/CMO was fully reconciled.

Unused IMP must not be discarded or used for any purpose other than the present trial. IMP that has been dispensed to a subject must not be re-dispensed to a different subject, unless agreed with the Sponsor.

A Trial Monitor will verify the IMP accountability forms.

6.9 Assessment of Investigational Medicinal Product Compliance

All IMPs will be administered as oral tablets dispersed in water (or put directly on the tongue without water in treatments A and B) under the supervision of the Investigator or designee. The IMP administration will be recorded in the CRF.

Drug administration records will be used to assess compliance.

The investigator is responsible for the control of drugs under investigation; adequate records of the receipt (e.g., Drug Receipt Record) and disposition (e.g., Drug Accountability Record, Drug

Destruction Record) of the investigational drug, including dates, quantities, and use by subject, must be maintained.

All records and drug supplies must be available for inspection at every monitoring visit. When the study is terminated, and drug accountability has been satisfactorily completed by the pharmacist/investigator or study drug preparer (or designee), the used and unused study drug (i.e., empty, partially used, and unused containers), will be returned to the Sponsor or its affiliate following the instructions given by the Sponsor. The completed Drug Accountability and Drug Destruction Records will be sent to the monitor or its designee.

The site pharmacist or study drug preparer must maintain records of the delivery of the IMP to the trial site, the inventory at the site, the use by each subject and its destruction or returning after drug accountability has been performed by the responsible monitor. The drug-dispensing log must be kept current, listing the identification of the subject who received drug along with the date and quantity of drug dispensed; it must be available for monitoring. Temperature of the storage refrigerator will be monitored and documented.

The investigator will ensure that the study drug supply is not used for any purpose other than this trial.

6.10 Method of Blinding

All oral suspension formulations will be prepared by study personnel (pharmacist) in another location than the area where the taste tests will be conducted. Immediately prior to tasting**,** a small amount of the assigned formulation will be drawn into a plastic syringe identified by a number. All oral suspensions will be drawn into identical syringes which have been wrapped with tape obscuring the content to minimize bias and blind the subjects and the investigator from the identity of the formulation.

All breaks of the trial blind must be adequately documented.

6.11 Emergency Unblinding

A study physician may request the pharmacist to unblind a volunteer in the case of a medical condition judged to be important enough for unblinding.

6.12 Treatment of Overdose

Not applicable as this is a swill and spit study.

6.13 Medical Care of Subjects after End of Trial

After the trial a therapeutic dose of Praziquantel will be made available to the local health council and management team to provide to the participating school according to local instructions. Normal medical care will follow the country specific regulations at the local clinic outside of the sponsor’s responsibility.

7 Trial Procedures and Assessments

7.1 Schedule of assessments

7.1.1 Screening procedures

During two weeks of the initial screening period all eligible study subject who are 6-11 years old will be identified in a primary school setting among pupils who are in class two up to class five. An informed consent will be obtained from the parents of the pupils that seem eligible to participate in the taste study. The pupils will be further asked an assent to participate in the study. Those pupils will then be trained to taste different flavour of juice, keep a candy in the mouth for 20 sec. without swallowing it and the use of hedonic scale for interpreting their feelings. Before the actual randomization all pupils will be further examined for their health status.

In order to be able to include 48 volunteers in the study some additional volunteers (from all sexes and age groups) will be asked to come back on Day 1 in morning at about 2 hour prior to the first taste test.

7.1.2 Study Day 1

On Day 1 a breakfast will be offered to all volunteers and after the physical examination, vital sign assessment and recheck of eligibility the randomization assignment will be done such that to balance the sequence of the palatability assessment in order that every formulation is tasted on an equal number of times between gender and the age groups.

According to gender and age, each subject will be randomized to receive a code to define the taste assessment sequence. These sequences will run from F_1 and G_1 for Day 1 tastes and H_2-N_2 all denoting permutations for tastes that will sequentially be performed on Day 2. In total there will be 5 taste assessments that will be assigned on both days. With the randomization step each volunteer will receive an enrolment number composed of 5 digits. The first two digits reflect the sex and age group and the last three digits incorporate the screening number.

The tablets (L-PZQ and Rac-PZQ ODTs) assessed on Day 1 will be put on the tongue (in the order given from the randomization) each to self-dispersion and at the end of each dispersion the subject will be asked to spit out the content.

Before every taste assessment the volunteer need to fast for about 1 hour. This period can be used to perform the physical examination, eligibility check and randomization.

The following steps will be performed for the first taste assessment:

1. **Around 5 min before the taste assessment all volunteers receive a cracker and rinse their mouth with a small cup of water.**

⬇

2. **Taste assessment (self-dispersion of tablets on the tongue, according to randomization sequence)**

⬇

3. **Immediately after the study drug has been spit out all volunteers will be asked to place a mark along the line with the use of a 100 mm visual analogue scale (VAS) that incorporates a 5 points hedonic scale for "overall palatability".**

⬇

4. **Between 2-5 minutes after the study drug has been spit out an open ended questionnaire (description of mouth feeling and taste description) will be conducted for each child and the child is asked again to place a mark along the 100 mm visual analogue scale (VAS)**

⬇

5. **Then all volunteers will be asked to wash their mouth with water and a cracker will be given to neutralize the taste.**

In addition, the study nurse will further record any discomfort or other observation in relation to acceptance of the study medication (e.g. spitting out of the medicine) that will be reported by the parents or investigator.

After a washout period of about 1h the child will be asked to assess the second treatment (according to the randomization scheme) and the steps 1-5 are repeated.

After the second taste assessment, the subject will be asked to remain at the study site for two additional hours for observation and during this time physical examination and vital sign assessment will be performed.

Subjects will be asked to come on the next day for the last taste assessments.

7.1.3 Study Day 2

On Day 2 similar exercise will be performed to mark a sequential flow that started on Day 1 with both tablets dispersed in water hence the subject is asked to hold a solution for 10 seconds in random order for each ingredient separately, and then to spit. Similarly the Praziquantel tablet will be crushed and then dispersed in water, and subsequently ¼ of the dispersion offered to each subject to test the taste. Day 2 will mark the end of the remaining three sequential tests resulting in a total of 5 taste periods as described in section 6.3.

When the volunteers show up on Day 2 about two hours before the first taste assessment a breakfast will be offered to them and a physical examination and vital sign assessment will be done. After a fasting period of about 1 hour the volunteer can then proceed with the first taste assessment.

1. **Around 5 min before the taste assessment all volunteers receive a cracker and rinse their mouth with a small cup of water.**

↓

2. **Taste assessment (dispersed/crushed in water, according to randomization sequence)**

↓

3. **Immediately after the study drug has been spit out all volunteers will be asked to place a mark along the line with the use of a 100 mm visual analogue scale (VAS) that incorporates a 5 points hedonic scale for "overall palatability".**

↓

4. **Between 2-5 minutes after the study drug has been spit out an open ended questionnaire (description of mouth feeling and taste description) will be conducted for each child and the child is asked again to place a mark along the 100 mm visual analogue scale (VAS)**

↓

5. **Then all volunteers will be asked to wash their mouth with water and a cracker will be given to neutralize the taste.**

In addition, as indicated for Day 1, the nurse will further record any discomfort or other observation in relation to acceptance of the study medication (e.g. spitting out of the medicine) that will be reported by the parents or investigator.

After a washout period of about 1h, the child will be asked to assess the second treatment (according to the randomization scheme) and the steps 1-5 are repeated.

About 1h after the second taste assessment, the child will be asked to assess the third treatment (according to the randomization scheme) and the steps 1-5 are repeated again.

After the last taste assessment, the subject will be asked to remain at the study site for two additional hours for observation and during this time a physical examination and vital sign assessment will be performed which are considered as the end of study assessment.

If an enrolled volunteer does not show up on Day 2 or for any reason cannot perform the taste assessments for all 5 treatment arms the volunteer will be replaced by a volunteer of same sex, and age group which will receive the same randomization code.

7.1.4 Post study

After the trial a therapeutic dose of Praziquantel will be made available to the local health council and management team to provide to the participating school.

7.1.5 Schedule of Assessments

	Screen -days																
Day/minutes related to IMP administration per period		Day 1							Day 2								
		Minutes							Minutes								
		-120 to 0	0	+2 to 5	60	+2 to 5		180	-120 to 0	0	+2to5	60	+2 to 5	120	+2 to 5		240 EOT
Administration of IMP (spill and spit)			X		X					X		X		X			
Confinement[1]																	
ICF signature	X																
Inclusion/exclusion criteria (re)checked	X	X							X								
Demographics, medical and medication history	X																
Physical examination[2]	X	X					X		X							X	
Vital signs (RR, HR, Temperature)	X	X					X		X							X	
VAS scale			X	X	X	X				X	X	X	X	X	X		
Open questionnaire				X		X					X		X		X		
Discharge to go home								X									X
AEs			X														

Figure 1- Schedule of assessment (detailed timing)

EOT: End-of-trial examination at two hours after last taste assessment

1.Confinement: From about 2h prior to first IMP administration on each day until about 2h after the last IMP of each day

2. Physical examination: According to study team standards

Prior to performing any trial assessments that are not part of routine medical care for the subject, the Investigator will obtain written informed consent as described in Section 9.2.

7.2 Demographic and Other Baseline Characteristics

The study should be done in children and a country where schistosomiasis is endemic and where WHO already donates the current formulation for prophylactic treatment. The choice of Tanzania was made due to the fact that schistosomiasis is endemic in this country and children there have usually been exposed to PZQ treatment (WHO program).

At screening, the following demographic data will be collected: date of birth, gender, weight, height.

The following relevant medical history data will be captured: previous illness, concomitant illness at entry into the trial, allergies and study relevant previous medications.

7.3 Palatability Assessments

All volunteers will be asked to place a mark along the line with the use of a 100 mm visual analogue scale (VAS) that incorporates a 5 points hedonic scale for “overall palatability” (see Questionnaire Appendix III).

In addition, any discomfort or other observation in relation to acceptance of the study medication (e.g. precipitated spitting out of the medicine) will be reported by the parents or investigator.

An open ended questionnaire (description of mouth feeling and taste description) will be conducted for each child during the washout period.

Recording of any discomfort or other observation will be done throughout the study.

7.4 Assessment of Safety

The safety profile of the IMP will be assessed through checking, baseline medical conditions, adverse events (AEs) and physical examination observations.

The investigator will report any AEs, whether observed by the Investigator or reported by the subject (see Section 7.8.1.2). The reporting period for AEs is from ICF signature until subject discharge at Day 2. Spontaneously reported adverse events (by parents/guardian) will be recorded in the CRF until one week after the last taste assessment.

7.4.1 Adverse Events

7.4.1.1 Adverse Event Definitions

Adverse Event

An AE is any untoward medical occurrence in a subject or clinical investigation subject administered a pharmaceutical product, regardless of causal relationship with this treatment. An AE can therefore be any unfavorable and unintended sign (including an abnormal laboratory finding), symptom, or disease temporally associated with the use of a medicinal product, whether or not considered related to the medicinal product.

For surgical or diagnostic procedures, the condition/illness leading to such a procedure is considered as the AE rather than the procedure itself.

The Investigator is required to grade the severity of each AE.

Accidental swallowing of the treatment (tablet or suspension) will be recorded in the CRF.

The investigators must assess the severity of AEs according to the Qualitative Toxicity Scale, as follows:

Mild: The subject is aware of the event or symptom, but the event or symptom is easily tolerated.

Moderate: The subject experiences sufficient discomfort to interfere with or reduce his or her usual level of activity.

Severe: Significant impairment of functioning: the subject is unable to carry out his or her usual activities.

Investigators must also systematically assess the causal relationship of AEs to IMPs using the following definitions. Decisive factors for the assessment of causal relationship of an AE to the IMPs include, but may not be limited to, temporal relationship between the AE and the IMP, known side effects of the IMPs, medical history, concomitant medication, course of the underlying disease, trial procedures.

Unrelated: Not reasonably related to the IMP/study treatment. AE could not medically (pharmacologically/clinically) be attributed to the IMP/study treatment under study in this clinical trial protocol. A reasonable alternative explanation must be available.

Related: Reasonably related to the IMP/study treatment. AE could medically (pharmacologically/clinically) be attributed to the IMP/study treatment under study in this clinical trial protocol.

Serious Adverse Events

An SAE is any untoward medical occurrence that at any dose:

- Results in death.
- Is life-threatening.

(Note: The term “life-threatening” refers to an event in which the subject is at risk of death at the time of the event, not an event that hypothetically might have caused death if it was more severe.)

- Requires inpatient hospitalization or prolongs an existing hospitalization.
- Results in persistent or significant disability or incapacity.
- Is a congenital anomaly or birth defect.
- Is otherwise considered as medically important.

(Note: Important medical events that may not result in death, be life-threatening, or require hospitalization may be considered as SAEs when, based upon appropriate medical judgment, they may jeopardize the subject or may require medical or surgical intervention to prevent one of the outcomes listed above. Examples of such events include allergic bronchospasm requiring intensive treatment in an emergency room or at home, blood dyscrasias or convulsions that do not result in inpatient hospitalization)

For the purposes of reporting, any suspected transmission of an infectious agent via an IMP is also considered an SAE, as described in 7.4.1.4

Events that Do Not Meet the Definition of an SAE

Elective hospitalizations to administer, or to simplify trial treatment or trial procedures (for example, an overnight stay to facilitate chemotherapy and related hydration therapy application) are not considered SAEs. However, all events leading to unplanned hospitalizations or unplanned prolongation of an elective hospitalization (for example, undesirable effects of any administered treatment) must be documented and reported as SAEs.

Events Not to Be Considered as AEs/SAEs

Medical conditions present at the initial trial visit that do not worsen in severity or frequency during the trial are defined as Baseline Medical Conditions, and are not to be considered AEs.

7.4.1.2 Methods of Recording and Assessing Adverse Events

On both visit days, the subject and the parents / representatives will be queried on changes in his or her condition by questioning. During the reporting period, any unfavorable changes in the subject’s condition will be recorded as AEs, whether reported by the subject or observed by the investigator.

Complete, accurate and consistent data on all AEs experienced for the duration of the reporting period (defined below) will be reported on an ongoing basis in the appropriate section of the CRF. All SAEs must be additionally documented and reported using the appropriate Serious Adverse Event Report Form as described in Section 7.4.1.4

It is important that each AE report include a description of the event, its duration (onset and resolution dates and times, its severity, its causal relationship with the trial treatment, any other potential causal factors, any treatment given or other action taken, and its outcome. In addition, serious cases should be identified and the appropriate seriousness criteria documented.

Specific guidance can be found in the CRF Completion and Monitoring Conventions provided by the project management.

An AE monitoring and reporting system for this study will be implemented according to study specific instructions developed based on this protocol.

7.4.1.3 Definition of the Adverse Event Reporting Period

The AE reporting period for safety surveillance begins when the subject is initially included in the trial (date of signature of informed consent) and continues until the visit of Day 2.

Any SAE assessed as related to the used IMPs must be reported whenever it occurs, irrespective of the time elapsed since the last administration of the IMP.

7.4.1.4 Procedure for Reporting Serious Adverse Events

Serious Adverse Events

In the event of any new SAE occurring during the reporting period, the Investigator must immediately (within a maximum of 24 HOURS after becoming aware of the event) inform the Sponsor or its designee and the Safety Monitoring Committee in writing.

All written reports should be transmitted using the SAE Report Form, which must be completed by the Investigator following specific completion instructions.

In exceptional circumstances, an SAE (or follow-up information) may be reported by telephone; In these cases, a written report must be sent immediately thereafter by fax or e-mail. Names, addresses, and telephone and fax numbers are for SAE reporting will be included in the trial-specific SAE Report Form and other instructions.

Relevant pages from the CRF may be provided in parallel (for example, medical history, concomitant drugs). Additional documents may be provided by the Investigator, if available (for example, laboratory results, hospital report and autopsy report). In all cases, the information provided on the SAE Report Form must be consistent with the data about the event recorded in the CRF.

The Investigator must respond to any request for follow-up information (for example, additional information, outcome, final evaluation, other records where needed) or to any question the Sponsor/designee may have on the AE within the same timelines as those noted above for initial reports. This is necessary to ensure prompt assessment of the event by the Sponsor or designee and (as applicable) to allow the Sponsor to meet strict regulatory timelines associated with expedited safety reporting obligations. Requests for follow-up will usually be made via the responsible Monitor, although in exceptional circumstances the Global Drug Safety department may contact the Investigator directly to obtain further information or to discuss the event.

7.4.1.5 Safety Reporting to Health Authorities, Independent Ethics Committees/ Institutional Review Boards and Investigators

The Investigator will send appropriate safety notifications to Health Authorities in accordance with applicable laws and regulations.

The Investigator must comply with any applicable site-specific requirements related to the reporting of SAEs (particularly deaths) involving trial subjects to the IEC/IRB that approved the trial.

In accordance with ICH GCP, the Sponsor/designee will inform the Investigator of “findings that could adversely affect the safety of subjects, impact the conduct of the trial or alter the IEC’s/IRB’s approval/favorable opinion to continue the trial.” In particular and in line with respective regulations, the Sponsor/designee will inform the Investigator of AEs that are both serious and unexpected and are considered to be related to the administered product (“suspected unexpected serious adverse reactions” or SUSARs). The Investigator should place copies of Safety Reports in the Investigator Site File. National regulations with regard to Safety Report notifications to Investigators will be taken into account.

All SAEs that are related to study participation (e.g. procedures, a change from existing therapy) or are related to a concurrent medication will be recorded from ICF signature until discharge

All SAE reports and/or all SAE participants-related safety information should be sent to Merck Serono Global Drug Safety Department:
GlobalDrugSafety@merckgroup.com
Telephone: +49 6151 72 8101
Fax: +49 6151 72 6914

The SAE will be summarized and reported to IRBs by the PI in the required time frame as specified by the IRB. The PI and/or the Sponsor will report the SAE to TFDA.

A written report of the SAE by the study site will be submitted within 24 hours for fatal and life threatening SAEs by phone, followed by paper reports within 7 calendar days of the investigator becoming aware of the SAE on the sponsor SAE report form, containing the minimal required information. All other cases should be reported within 14 days in order to comply with regulations mandating sponsor notification of specified SAEs to TFDA within 7 calendar days

When specifically required by regulations and guidelines, the Sponsor/designee will provide appropriate Safety Reports directly to the concerned lead IEC/IRB and will maintain records of these notifications. When direct reporting is not clearly defined by national or site-specific regulations, the Investigator will be responsible for promptly notifying the concerned IEC/IRB of any Safety Reports provided by the Sponsor/designee and of filing copies of all related correspondence in the Investigator Site File.

7.4.1.6 Monitoring of Subjects with Adverse Events

AEs are recorded and assessed continuously throughout the trial (see Section 7.4.1.3) and are assessed for final outcome at the Day 2. All SAEs ongoing must be monitored and followed up by the Investigator until stabilization or until the outcome is known, unless the subject is documented as “lost to follow-up”. Reasonable attempts to obtain this information must be made and documented. It is also the responsibility of the Investigator to ensure that any necessary additional therapeutic measures and follow-up procedures are performed.

7.4.2 Vital Signs, Physical Examinations, and Other Assessments

During screening and on Day 1 and Day 2 before the first and after the last taste assessment a physical examination and the vital signs as respiratory rate, heart rate and temperature are evaluated to make sure that the volunteer is able to do the taste assessment or at the end of the day can be discharged to go home.

Any abnormalities will be reported in the corresponding section in the CRF.

8 Statistics

A Statistical Analysis Plan (SAP) will provide full details of the analyses, data displays, and algorithms to be used for data derivations.

8.1 Sample Size

A total sample size of 48 subjects is considered adequate to fulfil the objective of the study.

Considering the design Day 1 (2 treatments), Day 2 (3 treatments) with the fact that the same subjects will be treated in both days, the stratification constraint by sex (female/male 1:1 ratio) and age ($<$9 yrs and $\geq$9yrs 1:1 ratio), the sample size should be a multiple of 24.

DAY 1: A total of 48 subjects (24 subjects per sequence) will provide at least 89.2% power to detect a 10 pts mean difference in VAS overall palatability between Rac-PZQ ODT (no water) and L-PZQ ODT (no water), assuming a within-subject standard deviation of 15 pts (square root of within subject MSE), and a two-sided type 1 error of 5.0%.

DAY 2: A total of 48 subjects (8 subjects per sequence, 6 sequences due to 3 treatments-3 periods) will provided at least 92.4% power to detect a 20 pts mean difference in VAS overall palatability between one of the new formulations Rac-PZQ (with water) or L-PZQ (with water) and the current PZQ formulation crushed in water, assuming a within-subject standard deviation

of 15 pts (square root of within subject MSE), and a two-sided type 1 error of 2.5%, to adjust for the multiplicity.

Software East 6.2 was used for the sample size calculation.

8.2 Randomization

Subjects will be randomized in blocks by sex (male/female 1:1 ratio) and age (<9 yrs and ≥9yrs 1:1 ratio) in one of the following treatment-sequence

Day 1: 2 treatments (no water) - 2 period cross-over

F1: Rac-PZQ ODT - L-PZQ ODT

G1: L-PZQ ODT - Rac-PZQ ODT

Day 2: 3 treatments (dispersed/crushed in water) - 3 period cross-over

H2: L-PZQ ODT - Rac-PZQ ODT- PZQ 150 mg

J2: L-PZQ ODT– PZQ 150 mg - Rac-PZQ ODT

K2: Rac-PZQ ODT– PZQ 150 mg - L-PZQ ODT

L2: Rac-PZQ ODT - L-PZQ ODT – PZQ 150 mg

M2: PZQ 150 mg - L-PZQ ODT- Rac-PZQ ODT

N2: PZQ 150 mg - Rac-PZQ ODT- L-PZQ ODT

The randomization list will be prepared by Swiss TPH.

8.3 Endpoints

8.3.1 Primary Endpoints

Overall palatability is assessed by putting a mark along the 100 mm visual analogue scale (VAS) that incorporates a 5-point hedonic scale and answering the questionnaire (see Annex III), and High score indicates good tasting.

The primary endpoint is the overall palatability VAS value taken at Time 0 (right after the spit-out of the IMP).

8.3.2 Secondary Endpoints

- Overall palatability VAS value at time 2-5min
- Description of mouth feeling and taste description using an open ended questionnaire

- Document discomfort and other observation in relation to acceptance of the study medication.

8.4 Analysis Sets

8.4.1 Full Analysis Set

The Full Analysis Set (FAS) includes all enrolled subjects assigned to study IMP, whether or not they received it.

8.4.2 Safety Set

The Safety Set (SAF) includes all enrolled subjects who received at least one dose of any study drug.

8.4.3 Completers Analysis Set

The Completers Analysis Set (CAS) includes all enrolled subjects who received at least one dose of any study drug and completed the entire study (Day 1 and Day 2).

8.5 Description of Statistical Analyses

8.5.1 General Considerations

All tabulations will be done by treatment group (current PZQ crushed and dispersed in water, Rac-PZQ ODT with water, L-PZQ ODT with water, Rac-PZQ ODT no water, L-PZQ ODT no water).

8.5.1.1 Subject Demographics

Baseline demographic and medical and medical history variables will be summarized for all subjects, overall and for each of the treatment group. For categorical variables, frequencies and percentages will be presented. Continuous variables will be summarized using descriptive statistics (mean, median, standard deviation, quartiles (25th, 75th), minimum, maximum).

8.5.1.2 Subject Accountability

The number of subjects who enrolled into the study, the number planned to be enrolled in each sequence, and the number and percentage of these subjects who were enrolled and completed each assessment will be presented. Frequency and percentage of subjects, who withdrew from the study, and reason for withdrawing, will be summarized overall and by treatment and treatment-sequence.

8.5.1.3 Vital signs and physical evaluation

Vital signs (Respiratory rates, heart rates and Temperature) and physical evaluation will be summarized for all subjects, overall and for each of the treatment group at each time point. For categorical variables, frequencies and percentages will be presented. Continuous variables using

value and change from baseline value will be summarized using descriptive statistics (mean, median, standard deviation, quartiles (25th, 75th), minimum, maximum).

8.5.2 Analysis of Primary Endpoints

VAS at time t0 will be summarized using descriptive statistics (mean, median, standard deviation, quartiles (25th, 75th), minimum, maximum) by treatment group.

The first alternative hypothesis (HA1) that the two formulations Rac-PZQ (no water) and L-PZQ (no water) differ in taste, as opposed to the H01 (the two formulations are similar) will be tested using a repeated measure ANOVA with factors time, sequence, treatment and stratification factor of sex and age (in categories). A contrast statement at Time 0 to compare the two formulation based on the likelihood ratio test will be conducted at two-sided 5% significance level.

The second alternative hypothesis (HA2) that at least one of the two new formulations (with water) differs in taste from the "current PZQ formulation" will be tested using a repeated measure ANOVA with factors time, sequence and treatment and stratification factor of sex and age (in categories) will be used.

Contrast statement at Time 0 to compare

- Rac-PZQ ODT (with water) vs current PZQ (crushed with water), and
- L-PZQ ODT (with water) vs current PZQ (crushed with water)

It will be done using the likelihood ratio test. These tests will be performed at an alpha level of 2.5% to account for the multiplicity of testing.

8.5.3 Analysis of Secondary Endpoints

All secondary endpoints will be performed at a significance level of 5% two-sided.

The analysis of the overall palatability will be described as secondary at time t2-5min.

8.5.4 Analysis of Safety and Other Endpoints

A treatment-emergent AE is any AE temporally associated with the use of study treatment, with onset date on or after initiation of study treatment until End of Day 2.

AEs will be coded using the MedDRA coding dictionary. All AEs will be displayed in subject listings. AEs will be categorized and tabulated by System Organ Class, Preferred Term, severity and relationship to IMP. Additionally listings of SAEs will be generated.

In the summary tables of subjects, if a subject has more than one occurrence of an AE for a specific Preferred Term, he/she will be counted only once for that preferred term. In the summary tables, the most severe occurrence of an AE, as well as the most extreme relationship

of the AE to the study treatment, will be indicated in cases of multiple occurrences of the same AE.

Adverse events summary will be displayed by treatment group for the following categories:

- All AEs
- Treatment-emergent adverse events
- Treatment emergent Serious adverse events
- Adverse events leading to study discontinuation

8.6 Safety Monitoring Committee (SMC)

For this study, a Safety Monitoring Committee will be appointed to follow up the safety of volunteers. The SMC will be composed of the following members:

- Merck Serono/EMD Serono Global Drug Safety (GDS) Product Lead (SMC Chairperson)
- Merck Serono/EMD Serono Medical Responsible
- Coordinating Investigator
- Independent Tanzanian expert

The members will be experienced clinicians and paediatricians qualified to evaluate safety data from this study. The SMC may be contacted for advice and independent review by the PI in the following situations:

- Following any SAE
- Any other situation where the Investigator or Sponsor thinks independent advice or review is important

9 Ethical and Regulatory Aspects

9.1 Responsibilities of the Investigator

The Investigator is responsible for the conduct of the trial at the site and will ensure that the trial is performed in accordance with this protocol, the ethical principles outlined in the Declaration of Helsinki, ICH GCP, and any other applicable regulations. The Investigator must ensure that only subjects who have given informed consent are included in the trial.

9.2 Subject Information and Informed Consent

An unconditional prerequisite for each subject prior to participation in the trial is written informed consent, which must be given before any trial-related activities are carried out. Adequate information must therefore be given to the subject by the Investigator or an appropriate designee (if local regulations permit) before informed consent is obtained.

A subject information sheet must be prepared in the local language in accordance with ICH GCP and will be provided by the Sponsor for the purpose of obtaining informed consent. In addition to providing this written information to a potential subject, the Investigator or a designate will inform the subject verbally of all pertinent aspects of the trial, using language chosen so that the information can be fully and readily understood by laypersons. The subject will be given sufficient time to read the information and the opportunity to ask questions and to request additional information and clarification.

If permitted by national regulations, a person other than the Investigator may inform the subject about the trial and sign the Informed Consent Form, as above.

After the information is provided by the Investigator, the Informed Consent Form must be signed and dated by the subject and the Investigator. The signed and dated declaration of informed consent will remain at the Investigator's site, and must be safely archived so that the forms can be retrieved at any time for monitoring, auditing and inspection purposes. A copy of the signed and dated information and Informed Consent Form should be provided to the subject prior to participation.

Whenever important new information becomes available that may be relevant to informed consent, the Investigator will revise the subject information sheet and any other written information to be provided to the subjects and submit them to the IRB for review and opinion. Using the approved revised subject information sheet and other written information, The Investigator will explain the changes to the previous version to each trial subject and obtain new written consent for continued participation in the trial. The subject will be given sufficient time to read the information and the opportunity to ask questions and to request additional information and clarification about the changes.

Written informed consent, in accordance with local practice, will provided by either parents or legal representative and in addition children will provide assent.

All Children will provide a separate written assent in addition to the parent consent.

9.3 Subject Identification and Privacy

A unique number will be assigned to each subject, immediately after informed consent has been obtained. This number will serve as the subject's identifier in the trial as well as in the clinical trial database. All subject data collected in the trial will be stored under the appropriate subject number. Only the Investigator will be able to link trial data to an individual subject via an identification list kept at the site. For each subject, original medical data will be accessible for the purposes of source data verification by the Monitor, audits and regulatory inspections, but patient confidentiality will be strictly maintained.

Data protection and privacy regulations will be observed in capturing, forwarding, processing, and storing subject data. Subjects will be informed accordingly, and will be requested to give their consent on data handling procedures in accordance with national regulations.

If the subject meets all inclusion criteria and does not meet any of the exclusion criteria, the investigator register the subject. If the subject is eligible for the trial, the subject number will be allocated and documented.

9.4 Emergency Medical Support and Subject Card

Generally, each subject will receive a card that includes certain minimum information pertaining to the trial, their participation, Investigator contact details and the appropriate trial unit for emergency medical support, and as applicable, for the process to unblind the treatment code.

Subjects will be provided with Emergency Medical Support cards for use during trial participation in order to provide clinical trial subjects with a way of identifying themselves as participating in a clinical trial and to give health care providers access to any information about this participation that may be needed to determine the course of medical treatment for the subject. The information provided on the Emergency Medical Support card may include the process for emergency unblinding.

The first point of contact for all emergencies will be the clinical trial Investigator caring for the affected subject. If the Investigator is available when an event occurs, they will answer any questions. Any subsequent action (for example, unblinding) will follow the standard process established for Investigators.

In cases where the Investigator is not available, the facility will provide the appropriate means to contact a physician.

9.5 Clinical Trial Insurance and Compensation to Subjects

Insurance conditions shall meet good local standards, as applicable.

The study centre has liability insurance, which is in accordance with Tanzanian regulations as guided by the Ministry of Health and Social Welfare.

The volunteers are compensated for their transport expenses as indicated in the informed consent form.

9.6 Independent Ethics Committee or Institutional Review Board

Prior to commencement of the trial at a given site, the project management shall be responsible for obtaining approval of the Ethics Committees (EKNZ in Basel, Switzerland, and IHI-IRB and NIMR in Tanzania) of the protocol and any subsequent amendments in compliance with local law before the start of the trial. The investigator(s) and sponsor's representatives will sign the protocol. The approval will be filed in the Investigator Site File. A copy will be filed in the Sponsor Trial Master File.

The Ethics Committees will be asked to document the date of the meeting at which the favourable opinion or approval was given and the members and voting members present.

Amendments to this clinical trial protocol will also be submitted to the concerned IRB, before implementation of substantial changes (see Section 10.5). Relevant safety information will be submitted to the IRB during the course of the trial in accordance with national regulations and requirements.

9.7 Health Authorities

The clinical trial protocol and any applicable documentation (for example, Investigational Medicinal Product Dossier, Subject Information and Informed Consent/Assent Forms) will be submitted or notified to the Health Authorities in accordance with all local and national regulations for each site.

10 Trial Management

10.1 Case Report Form Handling

Paper CRFs must be filled in completely and legibly, using either black or blue ballpoint pen suitable for use on official documents. Any necessary amendments or corrections must be made and countersigned and dated by the Investigator. When corrections are made to errors in data entry, the original entries must remain legible, and must not be deleted or obscured with correction aids. The Investigator must state his/her reasons for the correction of important data. For any missing data or remarks, the entry spaces provided in the paper CRF should be cancelled out to avoid unnecessary follow-up inquiries. The CRFs are essential trial documents and must be suitable for regulatory inspections and submissions.

10.2 Source Data and Subject Files

The Investigator must keep a file (medical file, original medical records) on paper for every subject in the trial. It must be possible to identify each subject by using this subject file. This file will contain the demographic and medical information for the subject listed below and should be as complete as possible.

- Subject’s full name, date of birth, sex, height, weight
- Medical history and concomitant diseases
- Prior and concomitant therapies (including changes during the trial)
- Trial identification, that is, the Sponsor trial number for this clinical trial, and subject number
- Dates for entry into the trial (informed consent) and visits to the site
- Any medical examinations and clinical findings predefined in this clinical trial protocol
- All AEs

- Date that the subject left the trial including any reason for early withdrawal from the trial or IMP (if applicable).

All documents containing source data must be filed, including, but not limited to CT or MRI scan images, ECG recordings, and laboratory results. Such documents must bear the subject number and the date of the procedure. If possible, this information should be printed by the instrument used to perform the assessment or measurement. As necessary, medical evaluation of such records should be performed; all evaluations should be documented, signed, and dated by the Investigator.

For data that may be recorded directly in the CRF such as a questionnaire or diary, there will be no record in the original subject file and therefore the data entered in the CRF will be considered source data.

10.3 Investigator Site File and Archiving

Upon initiation of the trial, the Investigator will be provided with an Investigator Site File containing all necessary trial documents, which will be completed throughout the trial and updated as necessary. The file must be available for review by the Monitor, during Sponsor audits and for inspection by Health Authorities during and after the trial, and must be safely archived for at least 15 years (or longer, per local requirements or as otherwise notified by the Sponsor) after the end of the trial. The documents to be archived include the Subject Identification List and the signed subject Informed Consent Forms. If archiving of the Investigator Site File is no longer possible at the site, the Investigator must notify the Sponsor/designee.

All original subject files (medical records) must be stored at the site (hospital, research institute, or practice) for the longest possible time permitted by the applicable regulations, and/or as per ICH GCP guidelines, whichever is longer. In any case, the Investigator should ensure that no destruction of medical records is performed without the written approval of the Sponsor.

10.4 Monitoring, Quality Assurance and Inspection by Health Authorities

This trial will be monitored in accordance with the ICH GCP, and any other applicable regulations. The site Monitor will perform visits to the trial site at regular intervals.

The clinical trial protocol, each step of the data capture procedure, and the handling of the data, including the final clinical trial report, will be subject to independent Quality Assurance activities. Audits may be conducted at any time during or after the trial to ensure the validity and integrity of the trial data. Representatives of the Quality Assurance unit from the Sponsor or a designated organization, as well as Health Authorities, must be permitted to access all trial documents and other materials at the site, including the Investigator Site File, the completed CRFs, all IMP and IMP accountability records, and the original medical records or files for each subject.

10.5 Changes to the Clinical Trial Protocol

Changes to the clinical trial protocol will be documented in writing. Substantive amendments will usually require submission to the Health Authorities and to the relevant IEC/IRB for approval or favourable opinion. In such cases, the amendment will be implemented only after approval or favourable opinion has been obtained.

Minor (nonsubstantial) protocol amendments, including administrative changes, will be filed by the Sponsor and at the site. They will be submitted to the relevant IEC/IRB or to Health Authorities only where requested by pertinent regulations. Any amendment that could affect the subject's agreement to participate in the trial requires additional informed consent prior to implementation following the process as described in Section 9.2.

10.6 Clinical Trial Report and Publication Policy

10.6.1 Clinical Trial Report

After completion of the trial, a clinical trial report will be written by the Sponsor in consultation with the Principal Investigator following the guidance in ICH Topic E3.

10.6.2 Publication

The first publication will include the results of the analysis of the primary endpoints. The Investigator will inform the Sponsor in advance about any plans to publish or present data from the trial. Any publications and presentations of the results (abstracts in journals or newspapers, oral presentations, etc.), either in whole or in part, by Investigators or their representatives will require review by the Sponsor before submission. The Sponsor will not suppress publication, but maintains the right to delay publication in order to protect intellectual property rights.

Appendix I: Signature Pages and Responsible Persons for the Trial

Signature Page – Protocol Lead

**Trial Title:**	Randomized, single blind, cross-over study to evaluate the palatability of new orally disintegrating tablets of PZQ and L-PZQ versus current PZQ tablets in African children age 6-11 years.
**Clinical Trial Protocol Date / Version:**	02.12.14 / V4.0

Protocol Lead responsible for designing the clinical trial:

I approve the design of the clinical trial:

[signature] 03 Dec 2014

Signature Date of Signature

Name, academic degree:	Wilhelmina Bagchus, PhD
Function / Title:	
Institution:	Institution EMD Serono R&D Institute<br>A subsidiary of Merck KGaA, Darmstadt, Germany
Address:	Address 45A Middlesex Turnpike, Billerica, MA 01821, USA
Telephone number:	Telephone number Phone: +1 978-294-1231
	Mobile: +1 978-435-1768
Fax number:	+1 978-294-1200
E-mail address:	wilhelmina.bagchus@emdserono.com

Signature Page – Principal Investigator

**Trial Title**	Randomized, single blind, cross-over study to evaluate the palatability of new orally disintegrating tablets of PZQ and L-PZQ versus current PZQ tablets in African children age 6-11 years.
**Clinical Trial Protocol Date / Version**	02.12.14 / V4.0
**Center Number**	1
**Principal Investigator**	Abdunoor Mulokozi Kabanywanyi

I, the undersigned, am responsible for the conduct of the trial at this site and affirm that I approve the design of the study and understand and will conduct the trial according to the clinical trial protocol, any approved protocol amendments, International Conference on Harmonisation Good Clinical Practice (Topic E6) and all applicable Health Authority requirements and national laws.

Signature: [signature]

Date of Signature: 02.12.2014

Name, academic degree: Abdunoor Mulokozi Kabanywanyi MD, PhD
Function / Title: Principal Investigator
Institution: Ifakara Health Institute
Address: Plot 463,Kiko Avenue
Mikocheni
PO Box 78373, Dar es Salaam, Tanzania
Telephone number: +255222774714
E-mail address: amulokozi@ihi.or.tz

Sponsor Responsible Person

Name, academic degree: Dr. Oezkan Yalkinoglu

Function / Title: Medical Responsible

Institution: Merck KGaA

Address: Frankfurter Strasse 250

64293 Darmstadt, Germany

Telephone number: +49 6151 72 29136

Fax number: +49 6151 72 3163

E-mail address: Email: oezkan.yalkinoglu@merckgroup.com

Appendix II: References

Works Cited

CHMP. (2005). *Reflection paper :formulation of choice for the paediatric population.* EMA.

Appendix III: Questionnaire

For each taste assessment, please have each subject complete the questionnaire with adult supervision.

The child answers the question by marking a line on the VAS scale line, as appropriate.

(i) Directly after spitting out the treatment

1 How did you like the taste of the medicine?

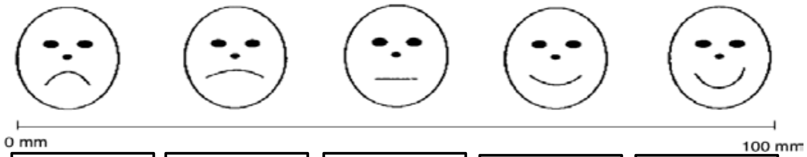

Very poor taste	Poor taste	Neither good nor bad	Good taste	Very good taste

(ii) 2-5 min after spitting out the treatment

2.1 How do you like the taste of the medicine now?

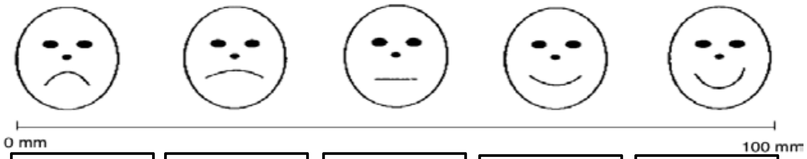

Very poor taste	Poor taste	Neither good nor bad	Good taste	Very good taste

2.2 Mark an X in the box next to ALL the words that best describe the taste of the medicine (e.g. sweet, bitter, sticky and smooth) inside your mouth?

❑ Sweet ❑ Sticky ❑ Bitter ❑ Smooth

❑ None of the above *(specify)*:___________________________________

Appendix IV: Conflict of interest statement

The Sponsor (Merck KGaA, Frankfurter Strasse 25064293, Darmstadt, Germany) and PZQ consortium are both working for a non-profit commitment to develop and register this treatment without intending to generate revenues for the company; therefore Merck declares no conflict of interest.
